# Associations of egg consumption with cardiovascular disease in a cohort study of 0.5 million Chinese adults

**DOI:** 10.1136/heartjnl-2017-312651

**Published:** 2018-05-21

**Authors:** Chenxi Qin, Jun Lv, Yu Guo, Zheng Bian, Jiahui Si, Ling Yang, Yiping Chen, Yonglin Zhou, Hao Zhang, Jianjun Liu, Junshi Chen, Zhengming Chen, Canqing Yu, Liming Li

**Affiliations:** 1 Department of Epidemiology and Biostatistics, School of Public Health, Peking University Health Science Center, Beijing, China; 2 Chinese Academy of Medical Sciences, Beijing, China; 3 Clinical Trial Service Unit and Epidemiological Studies Unit (CTSU), Nuffield Department of Population Health, University of Oxford, Oxford, UK; 4 Jiangsu Center for Disease Control and Prevention, Nanjing, China; 5 Liuyang Center for Disease Control and Prevention, Liuyang, China; 6 Jili Community Health Service, Liuyang, China; 7 China National Center for Food Safety Risk Assessment, Beijing, China

**Keywords:** egg consumption, cardiovascular disease, ischemic heart disease, major coronary events, hemorrhagic stroke, ischemic stroke, prospective study

## Abstract

**Objective:**

To examine the associations between egg consumption and cardiovascular disease (CVD), ischaemic heart disease (IHD), major coronary events (MCE), haemorrhagic stroke as well as ischaemic stroke.

**Methods:**

During 2004–2008, over 0.5 million adults aged 30–79 years were recruited from 10 diverse survey sites in China. Participants were asked about the frequency of egg consumption and were followed up via linkages to multiple registries and active investigation. Among 461 213 participants free of prior cancer, CVD and diabetes, a total of 83 977 CVD incident cases and 9985 CVD deaths were documented, as well as 5103 MCE. Stratified Cox regression was performed to yield adjusted hazard ratios for CVD endpoints associated with egg consumption.

**Results:**

At baseline, 13.1% of participants reported daily consumption (usual amount 0.76 egg/day) and 9.1% reported never or very rare consumption (usual amount 0.29 egg/day). Compared with non-consumers, daily egg consumption was associated with lower risk of CVD (HR 0.89, 95% CI 0.87 to 0.92). Corresponding multivariate-adjusted HRs (95% CI) for IHD, MCE, haemorrhagic stroke and ischaemic stroke were 0.88 (0.84 to 0.93), 0.86 (0.76 to 0.97), 0.74 (0.67 to 0.82) and 0.90 (0.85 to 0.95), respectively. There were significant dose-response relationships of egg consumption with morbidity of all CVD endpoints (P for linear trend <0.05). Daily consumers also had an 18% lower risk of CVD death and a 28% lower risk of haemorrhagic stroke death compared to non-consumers.

**Conclusion:**

Among Chinese adults, a moderate level of egg consumption (up to <1 egg/day) was significantly associated with lower risk of CVD, largely independent of other risk factors.

## Introduction

Cardiovascular disease (CVD) remains the leading cause of death and disability worldwide, including China, mostly due to ischaemic heart disease (IHD) and stroke (including both haemorrhagic and ischaemic stroke).^1^ Unlike IHD, which is the number 1 cause of premature death in most Western countries, stroke is the most responsible cause in China, followed by IHD. Although ischaemic stroke accounted for the majority of incident strokes, the proportion of incident haemorrhagic stroke in China was still higher than that in high-income countries (23.8% vs 9–13%).^2 3^

Modifiable factors, such as smoking, alcohol, physical inactivity and dietary factors, play crucial roles in the development of CVD, as do non-modifiable factors such as age and sex. Eggs are a prominent source of dietary cholesterol, but they also contain high-quality protein, many vitamins and bioactive components such as phospholipids and carotenoids.^4^ Previous studies have been inconsistent, and most of them observed insignificant associations between egg consumption and coronary heart disease (CHD) or stroke.^5^ However, the Life Span Study in Japan found that daily egg consumption was associated with a 30% lower risk of total stroke mortality compared with very occasional (rare) consumption.^6^ These studies had relatively smaller sample sizes or fewer cases, were too low-powered to obtain precise effect estimates, and were unable to examine the associations with stroke subtypes, especially haemorrhagic stroke. Above all, they originated from Western and Japanese populations, which have dietary habits and other lifestyle and CVD patterns that differ from the Chinese population. Dietary guidelines from the Chinese Nutrition Society recommend healthy adults consume 40–50 g of egg (about 0.8–1 egg) per day, with particular emphasis on the egg yolk, and recently cancelled the upper limit of cholesterol.^7^ Yet the amount of egg consumption has not improved over the past decade.^8^ Evidence about the relation between eggs and cardiovascular health among Chinese adults is therefore urgently required.

Our study aimed to examine the associations of egg consumption with CVD, including IHD, major coronary events (MCE), haemorrhagic stroke and ischaemic stroke in the China Kadoorie Biobank (CKB) study, an ongoing prospective cohort of about 0.5 million adults.

## Methods

### Study population

The CKB study was a prospective cohort of 512 891 participants aged 30–79 years from 10 geographically diverse survey sites (five urban and five rural) across China; participants were recruited from 2004 to 2008 and have been followed up to determine their morbidity and mortality rates ever since. About 5% of the participants were selected randomly to join in the re-surveys every 5 years after the completion of the baseline survey. Detailed descriptions of the CKB study have been previously published.^9 10^ In the present study, we excluded individuals reporting medical histories of cancer (n=2577), heart disease (n=15 472) or stroke (n=8884), or having prevalent diabetes (n=30 300) defined by self-reported diabetes or on-site plasma glucose testing (fasting blood glucose ≥7.0 mmol/L or random blood glucose ≥11.1 mmol/L). We made these exclusions to avoid a prevalence–incidence bias and minimise the effect of reverse causality led by potential confounders such as lifestyle factors. We also excluded two individuals whose body mass index (BMI) values were missing, and one individual with an implausible censoring date. Overall, 461 213 participants were eligible for the final analyses.

Both the Ethics Review Committee of the Chinese Center for Disease Control and Prevention (Beijing, China) and the Oxford Tropical Research Ethics Committee, University of Oxford (UK) have approved the CKB study. All participants signed informed consent forms before joining the study.

### Assessment of egg consumption

At baseline, we administered a laptop-based questionnaire to participants about the frequency of their habitual egg consumption during the previous 12 months at local clinics. Possible answers were daily, 4 to 6 days per week, 1 to 3 days per week, 1 to 3 days per month, and never or rarely. To evaluate the reproducibility of the baseline questionnaire, 926 participants were randomly chosen and interviewed repeatedly within a year (mean interval 5.4 months). The age- and sex-adjusted Spearman correlation coefficient for egg consumption was 0.58. Similar questions were asked at the first and second re-survey (2008 and 2013–14, respectively) and the amount was further provided during the second re-survey, which was used as a proxy to estimate the usual amount for each category (see the online supplementary material).

10.1136/heartjnl-2017-312651.supp1 Supplementary file 1

### Assessment of covariates

We collected covariates from the baseline questionnaire, including sociodemographic characteristics (age at recruitment, sex, education, household income, marital status), lifestyle behaviours (tobacco smoking, alcohol consumption, physical activity, intakes of multivitamin supplementation, rice, wheat products, other staple foods, meat, poultry, fish, dairy products, fresh vegetables, preserved vegetables, fresh fruit and soybean products), medical history (hypertension, diabetes, use of antihypertensive drugs, as well as use of aspirin and statins among those with self-reported hypertension), and family history of heart attack and stroke. A family history of CVD was considered to exist if a participant reported at least one first-degree relative (including biological parents and siblings) as having a heart attack or stroke. Three dietary patterns were identified previously and named as new affluence, traditional southern and traditional northern dietary pattern, respectively.^11^

Trained staff measured body weight, height, waist and hip circumferences, and blood pressure using calibrated instruments. BMI was calculated from the body weight in kilograms divided by the square of the height in metres (kg/m^2^). Waist to hip ratio was calculated from the waist circumference divided by the hip circumference. Prevalent hypertension was defined as self-reported diagnosed hypertension, a measured mean systolic blood pressure (SBP) ≥140 mm Hg, a measured mean diastolic blood pressure (DBP) ≥90 mm Hg, or self-reported use of antihypertensive drugs.

### Ascertainment of endpoints

Information on morbidity and mortality of all the participants was obtained regularly via local disease and death registries, checked against the national health insurance system with electronic linkage to all hospitalisations, or ascertained through active follow-up. Diseases were coded according to the International Classification of Diseases, 10th Revision (ICD-10). The primary outcomes were the incidence of and mortality from CVD (I00-I99), IHD (I20-I25), haemorrhagic stroke (I61) and ischaemic stroke (I63), as well as MCE, including fatal IHD death [I20-I25] and incident non-fatal myocardial infarction [I21-I23]).

### Statistical analysis

Logistic regression (for categorical variable) or multiple linear regression (for continuous variable) was conducted to compare age-, sex- and site-adjusted proportions or means of baseline characteristics by the frequency level of egg consumption.

We used stratified Cox proportional hazard models to estimate the hazard ratios (HRs) and 95% CIs for the associations between egg consumption and CVD, with stratification on survey site and birth cohort (in 5-year intervals) and with attained age as the underlying time scale. Person-years elapsed from completion of the baseline survey to the diagnosis of each CVD endpoint, death, loss to follow-up or 31 December 2015, whichever came first. The proportional hazard assumption was examined by testing the significance level of the interaction terms between egg consumption and time. The multivariate model was adjusted for age at recruitment (continuous), sex (men or women), education level (no formal school, primary school, middle school, high school, college, or university or higher), household income (<2500, 2500–4999, 5000–9999, 10 000–19 999, 20 000–34 999, or ≥35 000 yuan/year), marital status (married, widowed, divorced or separated, or never married), alcohol consumption (not weekly; ex-regular; not daily; daily consuming 1–15, 15–29, 30–59 or ≥60 g), tobacco smoking (never or occasional; former; current smoking with 1–14, 15–24, or ≥25 cigarettes/day), physical activity in MET-hours/day (continuous), BMI (continuous), waist to hip ratio (continuous), prevalent hypertension (presence or absence), use of aspirin (presence, absence, or unknown), family history of CVD (presence or absence), intake of multivitamin supplementation (presence or absence) and dietary pattern (new affluence, traditional northern, or traditional southern). We assigned the midpoint value to each frequency level and treated it as a continuous variable in the model to test the linear trend. The HR for each one egg increment per week was calculated with the usual amount of each frequency level in the model.

To examine the robustness of our results, we performed several sensitivity analyses: excluding cases or deaths occurring in the first 2 years of follow-up; additionally adjusting for daily energy intake^12^; additionally adjusting for the frequency levels of the other 11 food groups; adjusting for the dietary pattern without incorporating egg consumption, instead of the dietary pattern incorporating egg consumption; adjusting for simultaneous use of aspirin and statins instead of use of aspirin; adjusting for three levels of prevalent hypertension (absence, presence with antihypertensive medication, or presence without antihypertensive medication) instead of two levels of prevalent hypertension. We also examined the associations between egg consumption and CVD among diabetic patients.

We performed stratified analyses according to prespecified baseline groups: sex, age, area, education level, household income, alcohol consumption, tobacco smoking, level of physical activity, dietary pattern, BMI and hypertension. We examined the significance of interaction by the likelihood ratio test, comparing models with and without interaction terms between the stratifying variable and egg consumption (as a multinomial variable). All statistical analyses were performed with Stata version 14.0. We set the statistical significance at two-tailed P<0.05.

## Results

Among the eligible 461 213 participants, the mean age was 50.7 years; 41.0% were men; 42.3% resided in urban areas; about half of them consumed eggs 1–3 days per week. Participants consumed an average amount of 0.47 egg per day. Table 1 shows age-, sex- and site-adjusted baseline characteristics of the participants by category of egg consumption. Compared with non-consumers (those who never or rarely consumed eggs), daily consumers were more likely to have a higher level of education and household income, to have a new affluence dietary pattern, and to take multivitamin supplementation. Daily consumers were less likely to have prevalent hypertension compared with non-consumers.

**Table 1:** Baseline characteristics by category of egg consumption among 461 213 participants

Characteristics	Egg consumption					Overall (n=461 213)
	Never/rarely (n=42 046)	1–3 days/month (n=92 568)	1–3 days/week (n=2 16 990)	4–6 days/week (n=49 182)	7 days/week (n=60 427)	
Usual amount (egg/day)	0.29	0.36	0.46	0.56	0.76	0.47
Age (years)	52.3 (10.8)	51.2 (10.6)	50.2 (10.3)	49.7 (10.3)	51.6 (10.9)	50.7 (10.5)
Men (%)	33.9	38.6	42.2	42.3	44.2	41.0
Urban residence (%)	29.8	32.3	46.5	33.9	57.8	42.3
Highest education (%)						
Middle or high school	39.8	41.5	44.3	45.3	48.4	43.7
College and above	3.1	3.6	5.5	7.3	8.3	5.7
Household income (yuan/year, %)						
10 000–19 999	30.4	29.5	29.6	28.7	27.2	28.8
≥20 000	34.8	37.6	42.2	49.1	49.5	42.6
Married (%)	89.0	90.0	91.2	92.4	92.8	91.1
Current drinking (%)	19.3	17.9	18.6	18.5	21.0	15.2
Current smoking (%)	34.1	32.6	32.1	31.5	32.7	29.5
Physical activity (MET-hour/day)	21.5 (13.5)	22.1 (14.4)	21.9 (14.1)	21.9 (13.2)	21.7 (13.1)	21.9 (13.9)
Dietary pattern (%)						
New affluence	10.3	10.2	19.0	35.6	55.6	54.0
Traditional southern	64.6	64.1	56.6	41.0	23.7	23.5
Multivitamin supplementation (%)	2.7	2.6	3.4	4.4	6.5	3.7
BMI (kg/m^2^)	23.7 (3.5)	23.5 (3.3)	23.5 (3.3)	23.5 (3.3)	23.4 (3.4)	23.5 (3.3)
Hypertension (%)	36.9	34.1	32.2	30.5	29.0	32.4
Family history of CVD (%)	20.2	19.3	19.6	20.3	21.0	19.9

Values (except for the usual amount) according to the frequency of egg consumption were either proportion or mean (SD) with adjustment for age at recruitment, sex and survey site where appropriate, using logistic regression (for categorical variables) or multiple linear regression (for continuous variables). These variables were categorised into three levels: highest education level (primary school and below, middle or high school, or college and above), household income (<10 000, 10 000–19 999, or ≥20 000) and dietary pattern (new affluence, traditional southern or traditional northern).

BMI, body mass index; CVD, cardiovascular disease; MET, metabolic equivalent task.

During a median follow-up of 8.9 years (IQR 2.15 years; total person-years 3.9 million), we documented 83 977 CVD cases (including 30 169 IHD, 7078 haemorrhagic stroke, and 27 745 ischaemic stroke cases), 9985 CVD deaths (including 3374 IHD, 3435 haemorrhagic stroke, and 1003 ischaemic stroke deaths), as well as 5103 MCE. After multivariate adjustment, inverse associations were found to be significant between egg consumption and CVD (table 2). As compared with non-consumption, the adjusted HRs (95% CI) of daily consumption were 0.89 (0.87 to 0.92) for CVD, 0.88 (0.84 to 0.93) for IHD, 0.86 (0.76 to 0.97) for MCE, 0.74 (0.67 to 0.82) for haemorrhagic stroke, and 0.90 (0.85 to 0.95) for ischaemic stroke (all P for linear trend <0.05). Notably, each one egg increment per week was associated with an 8% lower risk of haemorrhagic stroke (HR 0.92, 95% CI 0.90 to 0.95). Similar associations were observed for CVD and haemorrhagic stroke mortality, with the HRs (daily consumption vs non-consumption) being 0.82 (0.75 to 0.89) and 0.72 (0.62 to 0.84), respectively (table 3). Yet the inverse associations with mortality from IHD and ischaemic stroke were found to be insignificant.

**Table 2:** Associations of egg consumption with risk of cardiovascular disease among 461 213 participants

Endpoints	Egg consumption					P for linear trend*	HR for 1 egg/week†
	Never/rarely	1–3 days/month	1–3 days/week	4–6 days/week	7 days/week		
CVD							
Cases	8125	17 086	38 147	8580	12 039		
Cases/PYs (1/1000)	22.7	21.9	20.8	20.8	23.6		
Model 1	1.00	0.94 (0.92 to 0.97)	0.88 (0.86 to 0.91)	0.86 (0.83 to 0.89)	0.84 (0.82 to 0.87)	<0.001	0.96 (0.95 to 0.96)
Model 2	1.00	0.97 (0.95 to 1.00)	0.92 (0.90 to 0.94)	0.90 (0.87 to 0.92)	0.89 (0.86 to 0.92)	<0.001	0.97 (0.96 to 0.97)
Model 3	1.00	0.97 (0.95 to 1.00)	0.92 (0.90 to 0.94)	0.90 (0.87 to 0.93)	0.89 (0.87 to 0.92)	<0.001	0.97 (0.96 to 0.98)
IHD							
Cases	2866	5529	13 541	3069	5164		
Cases/PYs (1/1000)	7.7	6.7	7.0	7.1	9.7		
Model 1	1.00	0.92 (0.88 to 0.97)	0.89 (0.86 to 0.93)	0.84 (0.80 to 0.88)	0.86 (0.82 to 0.90)	<0.001	0.96 (0.95 to 0.98)
Model 2	1.00	0.95 (0.91 to 0.99)	0.92 (0.88 to 0.96)	0.86 (0.82 to 0.91)	0.89 (0.85 to 0.93)	<0.001	0.97 (0.96 to 0.98)
Model 3	1.00	0.95 (0.91 to 0.99)	0.92 (0.88 to 0.96)	0.86 (0.81 to 0.91)	0.88 (0.84 to 0.93)	<0.001	0.97 (0.95 to 0.98)
MCE							
Cases	565	1033	2219	513	773		
Cases/PYs (1/1000)	1.5	1.2	1.1	1.2	1.4		
Model 1	1.00	0.89 (0.80 to 0.99)	0.86 (0.78 to 0.94)	0.74 (0.65 to 0.84)	0.75 (0.67 to 0.84)	<0.001	0.92 (0.89 to 0.95)
Model 2	1.00	0.93 (0.84 to 1.04)	0.93 (0.85 to 1.03)	0.83 (0.73 to 0.94)	0.85 (0.76 to 0.96)	0.001	0.95 (0.92 to 0.98)
Model 3	1.00	0.93 (0.84 to 1.04)	0.93 (0.85 to 1.03)	0.83 (0.73 to 0.94)	0.86 (0.76 to 0.97)	0.004	0.96 (0.93 to 0.99)
Haemorrhagic stroke							
Cases	953	1565	3080	757	723		
Cases/PYs (1/1000)	2.5	1.9	1.6	1.7	1.3		
Model 1	1.00	0.80 (0.73 to 0.86)	0.72 (0.67 to 0.78)	0.64 (0.58 to 0.71)	0.58 (0.52 to 0.64)	<0.001	0.86 (0.84 to 0.88)
Model 2	1.00	0.86 (0.79 to 0.93)	0.82 (0.76 to 0.88)	0.76 (0.68 to 0.84)	0.70 (0.63 to 0.78)	<0.001	0.91 (0.88 to 0.93)
Model 3	1.00	0.86 (0.79 to 0.93)	0.82 (0.76 to 0.88)	0.77 (0.70 to 0.86)	0.74 (0.67 to 0.82)	<0.001	0.92 (0.90 to 0.95)
Ischaemic stroke							
Cases	2840	5514	12 620	2613	4158		
Cases/PYs (1/1000)	7.6	6.7	6.5	6.0	7.7		
Model 1	1.00	0.95 (0.91 to 0.99)	0.91 (0.87 to 0.95)	0.88 (0.83 to 0.93)	0.81 (0.77 to 0.86)	<0.001	0.94 (0.93 to 0.96)
Model 2	1.00	0.98 (0.94 to 1.03)	0.95 (0.91 to 0.99)	0.94 (0.89 to 0.99)	0.88 (0.84 to 0.93)	<0.001	0.96 (0.95 to 0.98)
Model 3	1.00	0.98 (0.94 to 1.03)	0.95 (0.91 to 1.00)	0.95 (0.90 to 1.00)	0.90 (0.85 to 0.95)	<0.001	0.97 (0.96 to 0.98)

Stratified Cox proportional models were used with stratification on survey site and birth cohort (in 5-year intervals). Multivariate models were adjusted for: model 1: age at recruitment (continuous) and sex (men or women); model 2: additionally included education level (no formal school, primary school, middle school, high school, college, or university or higher), household income (<2500, 2500–4999, 5000–9999, 10 000–19,999, 20 000–34 999, or ≥35 000 yuan/year), marital status (married, widowed, divorced or separated, or never married), alcohol consumption (not weekly; ex-regular; not daily; daily consuming 1–15, 15–29, 30–59, or ≥60 g), tobacco smoking (never or occasional; former; current smoking with 1–14, 15–24, or ≥25 cigarettes/day), physical activity in MET-hours/day (continuous), BMI (continuous), waist to hip ratio (continuous), prevalent hypertension (presence or absence), use of aspirin (presence, absence, or unknown), family history of CVD (presence or absence); model 3: additionally included intake of multivitamin supplementation (presence or absence) and dietary pattern (new affluence, traditional northern, or traditional southern).

*Tests for linear trend were conducted by assigning 0, 0.5, 2.0, 5.0, 7.0 to the frequency levels from the lowest to the highest and treating the variable as a continuous variable in the Cox models.

†HR for each one egg increment per week were calculated by using the usual amount in the multivariate Cox models.

BMI, body mass index; CVD, cardiovascular disease; HR, hazard ratios; IHD, ischaemic heart disease; MCE, major coronary events; MET, metabolic equivalent task; PY, person-years.

**Table 3:** Associations of egg consumption with mortality from cardiovascular disease among 4 61 213 participants

Endpoints	Egg consumption					P for linear trend*	HR for 1 egg/week†
	Never/rarely	1–3 days/month	1–3 days/week	4–6 days/week	7 days/week		
PYs	382 204	838 613	1 970 423	442 564	551 465		
CVD							
Deaths	1316	2234	4296	935	1204		
Deaths/PYs (1/1000)	3.4	2.7	2.2	2.1	2.2		
Model 1	1.00	0.85 (0.80 to 0.91)	0.78 (0.73 to 0.83)	0.66 (0.60 to 0.72)	0.63 (0.58 to 0.69)	<0.001	0.87 (0.85 to 0.89)
Model 2	1.00	0.91 (0.85 to 0.98)	0.87 (0.82 to 0.93)	0.78 (0.71 to 0.85)	0.78 (0.71 to 0.84)	<0.001	0.93 (0.91 to 0.95)
Model 3	1.00	0.91 (0.85 to 0.98)	0.88 (0.82 to 0.94)	0.79 (0.73 to 0.87)	0.82 (0.75 to 0.89)	<0.001	0.94 (0.92 to 0.97)
IHD							
Deaths	395	664	1462	338	515		
Deaths/PYs (1/1000)	1.0	0.8	0.7	0.8	0.9		
Model 1	1.00	0.86 (0.75 to 0.97)	0.84 (0.75 to 0.94)	0.72 (0.62 to 0.84)	0.71 (0.62 to 0.82)	<0.001	0.91 (0.88 to 0.95)
Model 2	1.00	0.90 (0.79 to 1.02)	0.93 (0.83 to 1.05)	0.84 (0.72 to 0.97)	0.85 (0.74 to 0.97)	0.019	0.96 (0.92 to 0.99)
Model 3	1.00	0.90 (0.79 to 1.02)	0.94 (0.83 to 1.05)	0.85 (0.73 to 0.99)	0.88 (0.77 to 1.02)	0.131	0.97 (0.93 to 1.01)
Haemorrhagic stroke							
Deaths	540	842	1373	365	315		
Deaths/PYs (1/1000)	1.4	1.0	0.7	0.8	0.6		
Model 1	1.00	0.80 (0.72 to 0.89)	0.69 (0.62 to 0.76)	0.60 (0.52 to 0.69)	0.53 (0.46 to 0.62)	<0.001	0.83 (0.80 to 0.86)
Model 2	1.00	0.87 (0.78 to 0.97)	0.79 (0.71 to 0.88)	0.73 (0.63 to 0.84)	0.67 (0.58 to 0.78)	<0.001	0.89 (0.85 to 0.93)
Model 3	1.00	0.87 (0.78 to 0.97)	0.79 (0.71 to 0.88)	0.74 (0.65 to 0.86)	0.72 (0.62 to 0.84)	<0.001	0.91 (0.87 to 0.95)
Ischaemic stroke							
Deaths	113	237	437	90	126		
Deaths/PYs (1/1000)	0.3	0.3	0.2	0.2	0.2		
Model 1	1.00	0.97 (0.78 to 1.22)	0.90 (0.72 to 1.11)	0.82 (0.62 to 1.09)	0.73 (0.56 to 0.95)	0.006	0.90 (0.84 to 0.97)
Model 2	1.00	1.05 (0.83 to 1.31)	1.02 (0.82 to 1.27)	1.00 (0.75 to 1.34)	0.92 (0.70 to 1.21)	0.343	0.97 (0.90 to 1.04)
Model 3	1.00	1.05 (0.83 to 1.31)	1.02 (0.82 to 1.27)	1.00 (0.74 to 1.33)	0.93 (0.71 to 1.22)	0.388	0.97 (0.90 to 1.04)

Stratified Cox proportional models were used with stratification on survey site and birth cohort (in 5-year intervals). Multivariate models were adjusted for: model 1: age at recruitment (continuous) and sex (men or women); model 2: additionally included education level (no formal school, primary school, middle school, high school, college, or university or higher), household income (<2500, 2500–4999, 5000–9999, 10 000–19 999, 20 000–34 999, or ≥35 000 yuan/year), marital status (married, widowed, divorced or separated, or never married), alcohol consumption (not weekly; ex-regular; not daily; daily consuming 1–15, 15–29, 30–59, or ≥60 g), tobacco smoking (never or occasional; former; current smoking with 1–14, 15–24, or ≥25 cigarettes/day), physical activity in MET-hours/day (continuous), BMI (continuous), waist to hip ratio (continuous), prevalent hypertension (presence or absence), use of aspirin (presence, absence, or unknown), family history of CVD (presence or absence); model 3: additionally included intake of multivitamin supplementation (presence or absence) and dietary pattern (new affluence, traditional northern, or traditional southern).

*Tests for linear trend were conducted by assigning 0, 0.5, 2.0, 5.0, 7.0 to the frequency levels from the lowest to the highest and treating the variable as a continuous variable in the Cox model.

†HR for each one egg increment per week were calculated by using the usual amount in the multivariate Cox models.

BMI, body mass index; CVD, cardiovascular disease; HR, hazard ratios; IHD, ischaemic heart disease; MET, metabolic equivalent task; PY, person-years.

After excluding the events that occurred in the first 2 years of follow-up, the associations hardly changed; or there was additional adjustment for daily energy intake; or additional adjustment for the other 11 food groups; or adjustment for the dietary pattern without incorporating egg consumption, instead of the dietary pattern incorporating egg consumption; or adjustment for simultaneous use of aspirin and statins, instead of use of aspirin; or adjustment for three levels of prevalent hypertension, instead of two levels of prevalent hypertension (online supplementary tables S2–4). Further analyses demonstrated that egg consumption was not associated with morbidity and mortality of any CVD endpoint among diabetic patients (online supplementary tables S15–16).

**Figure 1:**
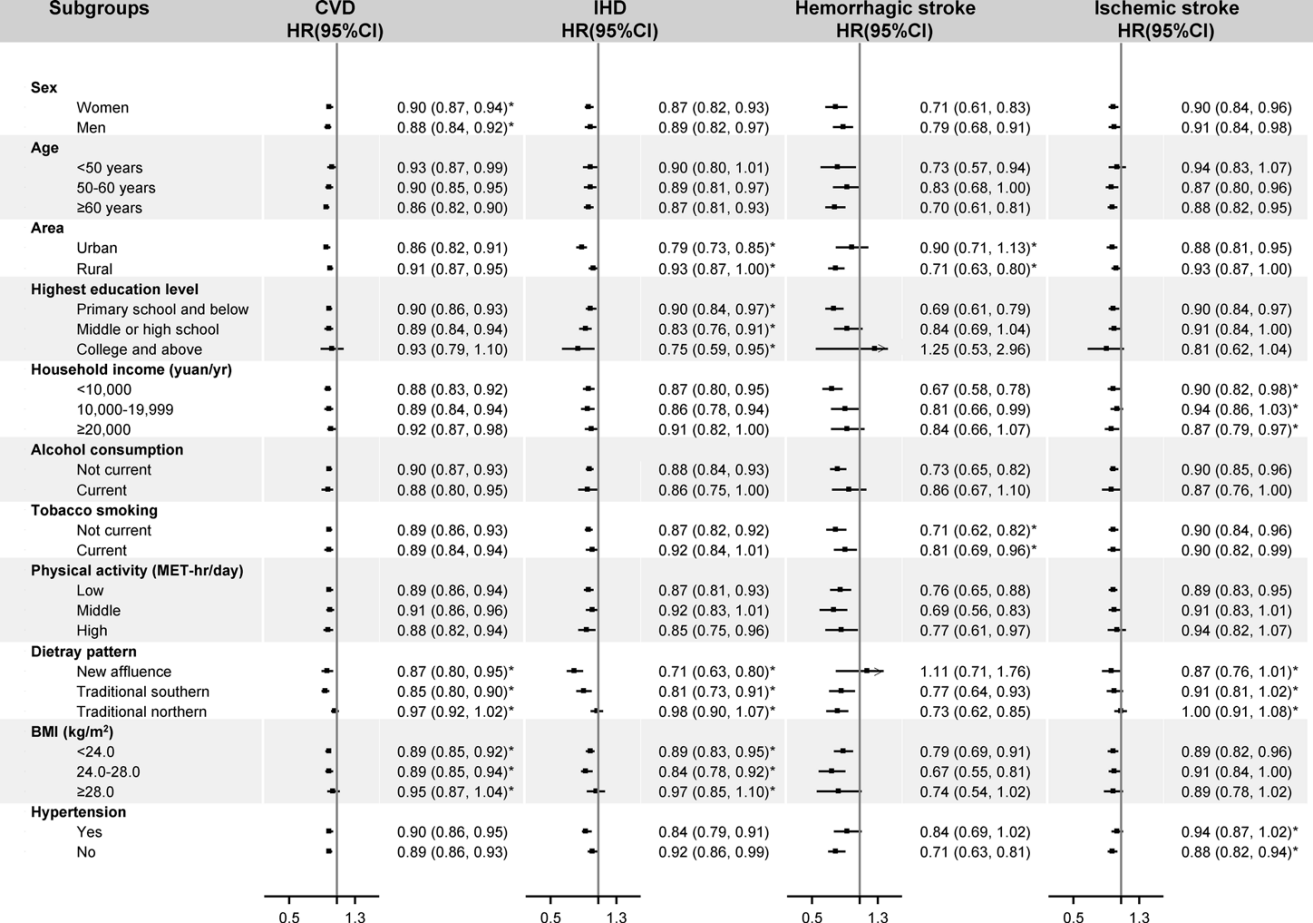
Subgroup analysis of associations between egg consumption and risk of incident cardiovascular disease (CVD), ischaemic heart disease (IHD), haemorrhagic stroke and ischaemic stroke according to potential baseline risk factors. Hazard ratios (HR) for incident CVD, IHD, haemorrhagic stroke and ischaemic stroke are for comparison between daily consumers and non-consumers. Solid squares represent point estimates, and horizontal lines represent 95% CI. Stratified Cox proportional models were used with stratification on survey site and birth cohort (in 5-year intervals). Multivariate models were adjusted for age at recruitment (continuous) and sex (men or women), education level (no formal school, primary school, middle school, high school, college, or university or higher), household income (<2500, 2500–4999, 5000–9999, 10 000–19 999, 20 000–34 999 or ≥35 000 yuan/year), marital status (married, widowed, divorced or separated, or never married), alcohol consumption (not weekly; ex-regular; not daily; daily consuming 1–15, 15–29, 30–59 or ≥60 g), tobacco smoking (never or occasional; former; current smoking with 1–14, 15–24 or ≥25 cigarettes/day), physical activity in MET-hours/day (continuous), BMI (continuous), waist to hip ratio (continuous), prevalent hypertension (presence or absence), use of aspirin (presence, absence or unknown), family history of CVD (presence or absence), intake of multivitamin supplementation (presence or absence) and dietary pattern (new affluence, traditional northern, or traditional southern). The tests for interaction were performed using likelihood ratio tests, which involved comparing models with and without interaction terms between the strata variable and egg consumption (as a multinomial variable). *Tests for interaction were significant, with the significant level being 0.05. BMI, body mass index; MET, metabolic equivalent task.

We conducted stratified analyses to examine whether the associations between egg consumption and CVD were modified by baseline characteristics. No significant interactions have been found across the stratum for age, alcohol consumption and physical activity (figure 1, online supplementary tables S6–14). Though significant interactions with sex were observed for incident CVD (P for interaction 0.005), the HRs were similar between men and women. We observed stronger associations with incident IHD among urban residents (P for interaction 0.014) and with haemorrhagic stroke among rural residents (P for interaction 0.048). For incident CVD and IHD, the associations appeared to be greater among participants with normal BMI (24.0–28.0 kg/m^2^) (P for interaction 0.005 and 0.046) or with traditional southern dietary pattern (P for interaction 0.031 and <0.001). Significant differences across the stratum were found, with stronger associations among non-current smokers than smokers (P for interaction 0.004). For daily consumers, inverse associations seemed to be greater among those with higher education level for IHD and those with higher household income for ischaemic stroke.

## Discussion

From this prospective cohort study, we found more frequent egg consumption was associated with CVD, IHD, MCE, haemorrhagic stroke and ischaemic stroke, independent of potential confounders. Notably, daily consumers (up to <1 egg/day) were associated with a 26% lower in risk of haemorrhagic stroke.

Results from our study have been inconsistent with previous studies. The latest meta-analysis included seven prospective studies and found that consumption up to one egg/day had no significant association with CHD (HR 0.97, 95% CI 0.88 to 1.07), compared with consuming two eggs/week.^5^ In the present study, a 12% reduction in risk of IHD was observed for consuming eggs daily (estimated amount 5.32 eggs/week), with never or rare consumption (2.03 eggs/week) as the reference group. This discrepancy may result from smaller sample sizes, fewer identified cases or deaths, ethnic difference, and only adjusting for prior diabetes or blood glucose instead of excluding diabetic patients. Their results may be biased by the inclusion of diabetic patients, who may alter their dietary habits and have a greater risk of CVD than non-diabetic patients.^13^ Our study excluded individuals with prevalent diabetes and sensitivity analyses further confirmed that the associations of interest appeared to attenuate null among diabetic patients.

The Life Span study in Japan documented 354 haemorrhagic stroke deaths and 655 ischaemic stroke deaths among 15 350 men (mean age 54 years) and 24 999 women (mean age 58 years) after 16 years of follow-up.^6^ They showed that the multivariate-adjusted HRs for total stroke mortality were 0.75 (95% CI 0.55–1.01) for less than one time/week, 0.77 (95% CI 0.57–1.03) for 2–4 times/week and 0.70 (95% CI 0.51–0.95) for almost daily consumption, compared with non-consumption (P for linear trend 0.185). Though another study from the Third National Health and Nutrition Examination Survey (NHANES III) found the inverse associations with stroke mortality, the estimate (HR 0.27, 95% CI 0.10 to 0.73) for daily consumption was imprecise because of sparse data (74 deaths among 6833 men and 8113 women).^14^ Both of the studies above failed to present the associations with stroke subtypes. The Nurses’ Health Study and Health Professionals Follow-Up Study analysed the associations with both haemorrhagic stroke and ischaemic stroke and observed that the three middle quintiles of egg consumption were associated with reduced risk of ischaemic stroke (from low to high: 16%, 14%, and 20%, respectively) compared with the lowest quintile category (P for linear trend 0.360).^15^ Other prospective studies did not observe similar associations between egg consumption and total stroke or any subtype.^16 17^ In the present study, higher egg consumption was associated with reduced risk of both stroke subtypes and participants consuming less than one egg daily were at the lowest risk of haemorrhagic stroke and ischaemic stroke compared with non-consumers.

Indeed, eggs are a major source of dietary cholesterol. However, a recent systemic review demonstrated that though dietary cholesterol increased the level of serum total cholesterol and low-density lipoprotein (LDL), it also elevated high-density lipoprotein (LDL) and the LDL to HDL ratio, and it had no established causal effect on CVD risk.^18^ Our findings of the inverse associations implicated that other components from eggs could have a favourable effect on cardiovascular health. Egg-derived phospholipid can raise HDL levels and enhance HDL function via preferentially incorporating into HDL cholesterol particles,^19^ further slowing down the progress of atherosclerosis.^20^ Likewise, high-quality egg protein resulted in greater satiety, lower postprandial glycaemia and insulinaemia, and reduced subsequent food intake in healthy and overweight individuals.^21 22^ Intake of eggs also increased plasma lutein and zeaxanthin,^23^ which play an important role in protecting against oxidation, inflammation and atherosclerosis.^24^ Notably, these two carotenoids derived from eggs had higher bioavailability than those derived from vegetables or fruits and promoted carotenoid absorption from other carotenoid-rich foods.^25 26^

The strengths of our study include a prospective cohort design, a large sample size, careful adjustment for established and potential risk factors for CVD, and the provision of more precise estimates for stroke subtypes. However, this study has some limitations. Egg consumption was obtained via a non-validated qualitative food frequency questionnaire and the average amounts were estimated indirectly from the two re-surveys, but the estimated amount was similar to that reported by the Chinese Nutrition and Health Surveillance (2010–2012: 0.49 egg).^8^ There is potential misclassification of egg consumption due to recall issues, but this misclassification may be non-differential, causing the associations to attenuate toward the null. Lacking participants with consumption of more than one egg per day restricted us to assess the association between higher egg consumption (>1 egg/day) and the risk of CVD; but the usual amount of the highest frequency level in the present study was approximate to the recommended amount of the guidelines (0.76 egg vs 0.8–1.0 egg), indicating that adherence to the dietary guidelines with regard to egg consumption could result in a lower risk of CVD. Another disadvantage is that information on habitual egg consumption was collected once at the the baseline survey and might not necessarily reflect dietary habits over the follow-up. Reverse causality could bias the results as well. Participants may change their habitual egg consumption after developing major chronic diseases or having diseases or subclinical symptoms that predispose them to CVD. However, we excluded participants with prior major diseases. Further exclusions of those developing CVD in the first 2 years of follow-up did not change the results appreciably. Finally, we controlled potential confounders to the best of our ability, yet residual confounding by other unmeasured or unknown factors may still exist.

## Conclusions

Our findings suggested that daily egg consumption (<1 egg) was associated with lower risk of CVD, IHD, MCE, haemorrhagic stroke and ischaemic stroke among Chinese middle-aged adults. Our findings contribute scientific evidence to the dietary guidelines with regard to egg consumption for the healthy Chinese adult.

**Key questions**

**What is already known on this subject?**

- The effect of egg consumption on cardiovascular disease (CVD) and stroke has been controversial. Previous studies mostly had smaller sample sizes, fewer CVD events and were unable to obtain accurate estimates on the associations of egg consumption with stroke subtypes.

**What might this study add?**

- This prospective cohort study of the general Chinese population demonstrated a significantly inverse association of egg consumption with CVD, ischaemic heart disease, haemorrhagic stroke, ischaemic stroke, as well as major coronary events. In particular, daily egg consumers (up to <1 egg/day) had a 26% lower risk of haemorrhagic stroke, a stroke subtype with a higher prevalence rate in China than in high-income countries.

**How might this impact on clinical practice?**

- The present study finds that there is an association between moderate level of egg consumption (up to <1 egg/day) and a lower cardiac event rate.
