# Supplementary material for: Associations of egg consumption with cardiovascular disease in a cohort study of 0.5 million Chinese adults
Source: Heart. 2018 May 21;104(21):1756–63. doi: 10.1136/heartjnl-2017-312651 (PMC6241631; doi:10.1136/heartjnl-2017-312651)
Supplement: Supplementary file 1 [file heartjnl-2017-312651supp001.docx]

**ONLINE SUPPLEMENT**

**Associations of Egg Consumption with Cardiovascular Disease in A Cohort Study of 0.5 Million Chinese Adults**

**Calculation of the usual amount of egg consumption**

The group average level of usual amount of egg consumption (in eggs per month), was calculated for each baseline consumption group, using the following steps:

1. The average daily amounts for each of the five egg consumption groups namely “never/rarely”, “1-3 days per month”, “1-3 days per week”, “4-6 days per week” or “daily” were computed using data collected during the 2nd resurvey. These were found to be 0 for the “never/rarely” group, 1.16 for the “1-3 days per month” group, 1.16 for the “1-3 days/week” group, 1.14 for the “4-6 days/week” group, and 1.17 for the “daily” group.

2. The average amounts per week of egg consumption were derived using the average daily amounts multiplied by the average number of days per month. The average number of days assigned to each group was 0 for the “never/rarely” group, 2 days for the “1-3 days per month” group, 8.57 days for the “1-3 days/week” group, 21.43 days for the “4-6 days/week” group, and 30 days for the “daily” group. Using these values lead to following estimates of average amounts per month of egg consumption of 0, 2.32, 9.94, 24.43, and 35.10, respectively, for “never/rarely”, “1-3 days per month”, “1-3 days per week”, “4-6 days per week” or “daily” egg consumption groups.

3. Assuming that the daily amounts of egg consumption did not vary much from the baseline to the 2nd resurvey (i.e. people may change their frequency of egg consumption but the daily amount of egg consumption remains roughly the same), the average amounts per month derived in the Step 2 was used as a proxy for the “baseline amount of egg consumption”.

4. The information from the 1st resurvey (Supplemental Table S1) was used to assess the variation in egg consumption from the baseline. The 1st resurvey was conducted at roughly the mid-point of the follow-up period. The average “usual amount of egg consumption” for each baseline group (“never/rarely”, “1-3 days per month”, “1-3 days per week”, “4-6 days per week” or “daily”) was then calculated by multiplying the “baseline amount of egg consumption” for each group (calculated in the Step 2) by the percentage of participants in each of the consumption frequency group at the 1st resurvey. For example, for the baseline “daily” group, at the 1st resurvey 45.93% remained at the “never/rarely” group, 14.94% switched to the “4-6 days per week” group, 30.83% switched to the “1-3 days per month” group, 4.23% switched to the “1-3 days per week” group, and 2.63% switched to the “never/rarely” group. The “usual amount of egg consumption” for the baseline “daily” group was therefore equal to 45.93%*35.10 + 14.94%*24.43 + 30.83%*9.94 + 4.23%*2.32 + 2.63%*0 = 22.72 eggs per month.

5. The “usual amount of egg consumption” for the other baseline categories of egg consumption (i.e. “never/rarely, “1-3 days per month”, “1-3 days per week”, or “4-6 days per week”) were calculated using the same approach, leading to 8.26, 10.66, 13.66 and 16.63 eggs per month respectively. This “usual amount of egg consumption” was then assigned to each individual participant in order to compute an effect size per 1 egg/week of egg consumption.

**Supplemental Table S1 Baseline and usual amount per month for each baseline category of egg consumption based on 19,788 participants who attended the first resurvey in 2008**

| Percentage (%)* | | 1st resurvey (mean 2.6 years later) **F** | | | | | Consumption days per month | Average daily amount†(eggs) | Baseline consumption (eggs/month) **B** | Usual consumption (eggs/month)‡ **U** |
| --- | --- | --- | --- | --- | --- | --- | --- | --- | --- | --- |
| Baseline | | Daily | 4-6 d/week | 1-3 d/week | 1-3 days/month | Never/rarely |  |  |  |  |
| n | | 1 | 2 | 3 | 4 | 5 |  |  |  |  |
| 1 | Daily | 45.93 | 14.94 | 30.83 | 5.67 | 2.63 | 30.00 | 1.17 | B_1_=35.10 | U_1_=22.72 |
| 2 | 4-6 d/week | 17.26 | 24.80 | 44.74 | 10.21 | 2.99 | 21.43 | 1.14 | B_2_=24.43 | U_2_=16.63 |
| 3 | 1-3 d/week | 10.75 | 17.06 | 56.24 | 11.90 | 4.05 | 8.57 | 1.16 | B_3_=9.94 | U_3_=13.66 |
| 4 | 1-3 days/month | 6.56 | 11.57 | 52.14 | 19.99 | 9.73 | 2.00 | 1.16 | B_4_=2.32 | U_4_=10.66 |
| 5 | Never/rarely | 7.24 | 6.98 | 35.73 | 23.60 | 26.45 | 0 | 0 | B_5_=0 | U_5_=8.26 |

* The percentage of each frequency level in the 1st resurvey was calculated according to five frequency levels in the baseline survey.

† The average daily amount number came from the 2nd resurvey data, used as a proxy of baseline mean daily portion.

‡ Usual intake amount for each group was estimated by taking into account changes in consumption frequency between baseline and 1st resurvey using this formula U_n_ = $\sum_{i=1}^{5} (F\mathrm{ni} \times Bi)$; F is the percentage in each cell, B is the baseline consumption amount per month for each baseline category, U is the usual consumption amount per month for each baseline category.

**Supplemental Table S2. Associations of egg consumption with risk of cardiovascular disease after excluding cases during the first 2 years of follow-up.**

| **Endpoints** | **Egg consumption** | | | | | **P for linear trend*** | **HR for 1 egg/week**† |
| --- | --- | --- | --- | --- | --- | --- | --- |
|  | **Never/rarely** | **1-3 days/month** | **1-3 d/week** | **4-6 d/week** | **7 d/week** |  |  |
| **CVD** |  |  |  |  |  |  |  |
| Cases | 7,148 | 14,381 | 32,194 | 7,402 | 10,380 |  |  |
| Cases/PYs (1000) | 20.1 | 18.5 | 17.6 | 18.0 | 20.4 |  |  |
| Model 1 | 1.00 | 0.94 (0.92, 0.97) | 0.88 (0.86, 0.91) | 0.86 (0.83, 0.89) | 0.85 (0.82, 0.87) | <0.001 | 0.96 (0.95, 0.97) |
| Model 2 | 1.00 | 0.97 (0.94, 1.00) | 0.92 (0.89, 0.94) | 0.90 (0.87, 0.94) | 0.90 (0.87, 0.93) | <0.001 | 0.97 (0.96, 0.98) |
| Model 3 | 1.00 | 0.97 (0.94, 1.00) | 0.92 (0.90, 0.95) | 0.91 (0.88, 0.94) | 0.90 (0.87, 0.93) | <0.001 | 0.97 (0.96, 0.98) |
| **IHD** |  |  |  |  |  |  |  |
| Cases | 2,534 | 4,626 | 11,352 | 2,622 | 4,399 |  |  |
| Cases/PYs (1000) | 6.8 | 5.7 | 5.9 | 6.1 | 8.3 |  |  |
| Model 1 | 1.00 | 0.91 (0.87, 0.96) | 0.89 (0.85, 0.93) | 0.84 (0.79, 0.89) | 0.86 (0.82, 0.91) | <0.001 | 0.97 (0.95, 0.98) |
| Model 2 | 1.00 | 0.94 (0.89, 0.99) | 0.92 (0.88, 0.96) | 0.87 (0.82, 0.92) | 0.90 (0.85, 0.94) | <0.001 | 0.97 (0.96, 0.99) |
| Model 3 | 1.00 | 0.94 (0.90, 0.99) | 0.92 (0.88, 0.96) | 0.86 (0.81, 0.91) | 0.89 (0.84, 0.94) | <0.001 | 0.97 (0.96, 0.98) |
| **MCE** |  |  |  |  |  |  |  |
| Cases | 493 | 895 | 1,949 | 463 | 698 |  |  |
| Cases/PYs (1000) | 1.3 | 1.1 | 1.0 | 1.0 | 1.3 |  |  |
| Model 1 | 1.00 | 0.89 (0.79, 0.99) | 0.85 (0.77, 0.95) | 0.76 (0.66, 0.87) | 0.77 (0.68, 0.87) | <0.001 | 0.93 (0.90, 0.96) |
| Model 2 | 1.00 | 0.93 (0.83, 1.04) | 0.93 (0.83, 1.03) | 0.84 (0.74, 0.96) | 0.87 (0.77, 0.98) | 0.016 | 0.96 (0.93, 0.99) |
| Model 3 | 1.00 | 0.93 (0.83, 1.04) | 0.93 (0.84, 1.03) | 0.85 (0.74, 0.97) | 0.88 (0.77, 1.00) | 0.032 | 0.97 (0.93, 1.00) |
| **Hemorrhagic stroke** |  |  |  |  |  |  |  |
| Cases | 805 | 1,317 | 2,651 | 633 | 603 |  |  |
| Cases/PYs (1000) | 2.1 | 1.6 | 1.4 | 1.4 | 1.1 |  |  |
| Model 1 | 1.00 | 0.79 (0.72, 0.87) | 0.72 (0.67, 0.79) | 0.63 (0.57, 0.71) | 0.57 (0.51, 0.64) | <0.001 | 0.85 (0.83, 0.88) |
| Model 2 | 1.00 | 0.85 (0.78, 0.93) | 0.82 (0.75, 0.89) | 0.74 (0.66, 0.83) | 0.69 (0.62, 0.77) | <0.001 | 0.90 (0.88, 0.93) |
| Model 3 | 1.00 | 0.85 (0.78, 0.93) | 0.82 (0.75, 0.89) | 0.76 (0.68, 0.85) | 0.73 (0.65, 0.82) | <0.001 | 0.92 (0.89, 0.95) |
| **Ischemic stroke** |  |  |  |  |  |  |  |
| Cases | 2,597 | 4,981 | 11,251 | 2,317 | 3,715 |  |  |
| Cases/PYs (1000) | 7.0 | 6.1 | 5.8 | 5.4 | 6.9 |  |  |
| Model 1 | 1.00 | 0.95 (0.91, 1.00) | 0.90 (0.86, 0.94) | 0.87 (0.82, 0.92) | 0.81 (0.77, 0.85) | <0.001 | 0.94 (0.93, 0.95) |
| Model 2 | 1.00 | 0.98 (0.93, 1.03) | 0.95 (0.90, 0.99) | 0.93 (0.87, 0.98) | 0.87 (0.83, 0.92) | <0.001 | 0.96 (0.95, 0.97) |
| Model 3 | 1.00 | 0.98 (0.93, 1.03) | 0.95 (0.91, 0.99) | 0.94 (0.89, 1.00) | 0.90 (0.85, 0.95) | <0.001 | 0.97 (0.96, 0.98) |

PY: person-years; CVD: cardiovascular disease; IHD: ischemic heart disease; MCE: major coronary events.

Stratified Cox proportional models were used with stratification on survey site and birth cohort (in 5-year intervals). Multivariate models were adjusted for age at recruitment (continuous) and sex (men or women); model 2: additionally included education level (no formal school, primary school, middle school, high school, college, or university or higher), household income (<2,500, 2,500-4,999, 5,000-9,999, 10,000-19,999, 20,000-34,999, or ≥35,000 yuan/year), marital status (married, widowed, divorced or separated, or never married), alcohol consumption (not weekly; ex-regular; not daily; daily consuming 1-15, 15-29, 30-59, or ≥60 g), tobacco smoking (never or occasional; former; current smoking with 1-14, 15-24, or ≥25 cigarettes/day), physical activity in MET-hours/day (continuous), BMI (continuous), waist to hip ratio (continuous), prevalent hypertension (presence or absence), use of aspirin (presence, absence, or unknown), family history of CVD (presence or absence); model 3: additionally included intake of multivitamin supplementation (presence or absence) and dietary pattern (new affluence, traditional northern, or traditional southern).

* Tests for linear trend were conducted by assigning 0, 0.5, 2.0, 5.0, 7.0 to the frequency levels from the lowest to the highest and treating the variable as a continuous variable in the Cox models.

† HRs for each 1 egg increment per week were calculated by using the usual amount in the multivariate Cox models.

**Supplemental Table S3. Associations of egg consumption with mortality from cardiovascular disease after excluding deaths during the first 2 years of follow-up.**

| **Endpoints** | **Egg consumption** | | | | | **P for linear trend*** | **HR for 1 egg/week**† |
| --- | --- | --- | --- | --- | --- | --- | --- |
|  | **Never/rarely** | **1-3 days/month** | **1-3 d/week** | **4-6 d/week** | **7 d/week** |  |  |
| **CVD** |  |  |  |  |  |  |  |
| Cases | 1,132 | 1,962 | 3,793 | 823 | 1,075 |  |  |
| Cases/PYs (1000) | 3.0 | 2.3 | 1.9 | 1.9 | 2.0 |  |  |
| Model 1 | 1.00 | 0.87 (0.81, 0.94) | 0.79 (0.74, 0.85) | 0.68 (0.62, 0.74) | 0.65 (0.60, 0.72) | <0.001 | 0.88 (0.86, 0.90) |
| Model 2 | 1.00 | 0.93 (0.86, 1.00) | 0.89 (0.83, 0.95) | 0.80 (0.73, 0.88) | 0.80 (0.73, 0.87) | <0.001 | 0.93 (0.91, 0.96) |
| Model 3 | 1.00 | 0.93 (0.86, 1.00) | 0.89 (0.83, 0.96) | 0.82 (0.74, 0.90) | 0.84 (0.77, 0.92) | <0.001 | 0.95 (0.93, 0.98) |
| **IHD** |  |  |  |  |  |  |  |
| Cases | 342 | 586 | 1,299 | 307 | 462 |  |  |
| Cases/PYs (1000) | 0.9 | 0.7 | 0.7 | 0.7 | 0.8 |  |  |
| Model 1 | 1.00 | 0.88 (0.77, 1.01) | 0.87 (0.76, 0.98) | 0.77 (0.65, 0.90) | 0.74 (0.64, 0.86) | <0.001 | 0.92 (0.89, 0.96) |
| Model 2 | 1.00 | 0.93 (0.81, 1.06) | 0.96 (0.85, 1.09) | 0.89 (0.76, 1.04) | 0.88 (0.76, 1.02) | 0.089 | 0.97 (0.93, 1.01) |
| Model 3 | 1.00 | 0.92 (0.81, 1.06) | 0.96 (0.85, 1.09) | 0.91 (0.77, 1.07) | 0.92 (0.79, 1.07) | 0.347 | 0.98 (0.94, 1.02) |
| **Hemorrhagic stroke** |  |  |  |  |  |  |  |
| Cases | 447 | 706 | 1,170 | 311 | 265 |  |  |
| Cases/PYs (1000) | 1.2 | 0.8 | 0.6 | 0.7 | 0.5 |  |  |
| Model 1 | 1.00 | 0.81 (0.72, 0.91) | 0.70 (0.62, 0.79) | 0.62 (0.53, 0.72) | 0.54 (0.46, 0.63) | <0.001 | 0.83 (0.80, 0.87) |
| Model 2 | 1.00 | 0.88 (0.78, 0.99) | 0.80 (0.71, 0.90) | 0.74 (0.64, 0.87) | 0.68 (0.58, 0.80) | <0.001 | 0.89 (0.85, 0.93) |
| Model 3 | 1.00 | 0.88 (0.78, 0.99) | 0.80 (0.72, 0.90) | 0.76 (0.65, 0.89) | 0.74 (0.63, 0.87) | <0.001 | 0.91 (0.87, 0.96) |
| **Ischemic stroke** |  |  |  |  |  |  |  |
| Cases | 100 | 214 | 397 | 76 | 116 |  |  |
| Cases/PYs (1000) | 0.3 | 0.3 | 0.2 | 0.2 | 0.2 |  |  |
| Model 1 | 1.00 | 1.00 (0.79, 1.28) | 0.92 (0.73, 1.15) | 0.79 (0.58, 1.08) | 0.76 (0.57, 1.00) | <0.001 | 0.91 (0.84, 0.98) |
| Model 2 | 1.00 | 1.07 (0.84, 1.36) | 1.04 (0.82, 1.31) | 0.95 (0.70, 1.30) | 0.94 (0.70, 1.24) | 0.288 | 0.96 (0.90, 1.04) |
| Model 3 | 1.00 | 1.07 (0.84, 1.36) | 1.04 (0.83, 1.31) | 0.96 (0.70, 1.31) | 0.96 (0.72, 1.29) | 0.418 | 0.97 (0.90, 1.05) |

PY: person-years; CVD: cardiovascular disease; IHD: ischemic heart disease.

Stratified Cox proportional models were used with stratification on survey site and birth cohort (in 5-year intervals). Multivariate models were adjusted for age at recruitment (continuous) and sex (men or women); model 2: additionally included education level (no formal school, primary school, middle school, high school, college, or university or higher), household income (<2,500, 2,500-4,999, 5,000-9,999, 10,000-19,999, 20,000-34,999, or ≥35,000 yuan/year), marital status (married, widowed, divorced or separated, or never married), alcohol consumption (not weekly; ex-regular; not daily; daily consuming 1-15, 15-29, 30-59, or ≥60 g), tobacco smoking (never or occasional; former; current smoking with 1-14, 15-24, or ≥25 cigarettes/day), physical activity in MET-hours/day (continuous), BMI (continuous), waist to hip ratio (continuous), prevalent hypertension (presence or absence), use of aspirin (presence, absence, or unknown), family history of CVD (presence or absence); model 3: additionally included intake of multivitamin supplementation (presence or absence) and dietary pattern (new affluence, traditional northern, or traditional southern).

* Tests for linear trend were conducted by assigning 0, 0.5, 2.0, 5.0, 7.0 to the frequency levels from the lowest to the highest and treating the variable as a continuous variable in the Cox models.

† HRs for each 1 egg increment per week were calculated by using the usual amount in the multivariate Cox models.

**Supplemental Table S4. Associations of egg consumption with risk of cardiovascular disease after further adjustment.**

| **Endpoints** | **Egg consumption** | | | | | **P for linear trend*** | **HR for 1 egg/week**† |
| --- | --- | --- | --- | --- | --- | --- | --- |
|  | **Never/rarely** | **1-3 days/month** | **1-3 d/week** | **4-6 d/week** | **7 d/week** |  |  |
| **CVD** |  |  |  |  |  |  |  |
| Basic model | 1.00 | 0.97 (0.95, 1.00) | 0.92 (0.90, 0.94) | 0.90 (0.87, 0.93) | 0.89 (0.87, 0.92) | <0.001 | 0.97 (0.96, 0.98) |
| Model 4 | 1.00 | 0.97 (0.95, 1.00) | 0.92 (0.90, 0.95) | 0.90 (0.87, 0.93) | 0.90 (0.87, 0.93) | <0.001 | 0.97 (0.96, 0.98) |
| Model 5 | 1.00 | 0.97 (0.95, 1.00) | 0.93 (0.90, 0.95) | 0.90 (0.87, 0.93) | 0.89 (0.87, 0.92) | <0.001 | 0.97 (0.96, 0.98) |
| Model 6 | 1.00 | 0.97 (0.95, 1.00) | 0.92 (0.90, 0.95) | 0.90 (0.87, 0.93) | 0.90 (0.87, 0.93) | <0.001 | 0.97 (0.96, 0.98) |
| Model 7 | 1.00 | 0.97 (0.95, 1.00) | 0.92 (0.90, 0.94) | 0.90 (0.87, 0.93) | 0.89 (0.87, 0.92) | <0.001 | 0.97 (0.96, 0.98) |
| Model 8 | 1.00 | 0.97 (0.95, 1.00) | 0.92 (0.90, 0.95) | 0.90 (0.87, 0.93) | 0.89 (0.87, 0.92) | <0.001 | 0.97 (0.96, 0.98) |
| **IHD** |  |  |  |  |  |  |  |
| Basic model | 1.00 | 0.95 (0.91, 0.99) | 0.92 (0.88, 0.96) | 0.86 (0.81, 0.91) | 0.88 (0.84, 0.93) | <0.001 | 0.97 (0.95, 0.98) |
| Model 4 | 1.00 | 0.95 (0.91, 0.99) | 0.92 (0.88, 0.96) | 0.86 (0.81, 0.91) | 0.88 (0.84, 0.93) | <0.001 | 0.97 (0.95, 0.98) |
| Model 5 | 1.00 | 0.95 (0.91, 0.99) | 0.93 (0.89, 0.97) | 0.86 (0.82, 0.91) | 0.88 (0.84, 0.93) | <0.001 | 0.97 (0.95, 0.98) |
| Model 6 | 1.00 | 0.95 (0.91, 0.99) | 0.92 (0.88, 0.96) | 0.86 (0.82, 0.91) | 0.89 (0.84, 0.93) | <0.001 | 0.97 (0.96, 0.98) |
| Model 7 | 1.00 | 0.95 (0.91, 0.99) | 0.92 (0.88, 0.96) | 0.86 (0.81, 0.91) | 0.88 (0.84, 0.93) | <0.001 | 0.97 (0.95, 0.98) |
| Model 8 | 1.00 | 0.95 (0.91, 1.00) | 0.92 (0.88, 0.96) | 0.86 (0.82, 0.91) | 0.88 (0.84, 0.93) | <0.001 | 0.97 (0.95, 0.98) |
| **MCE** |  |  |  |  |  |  |  |
| Basic model | 1.00 | 0.93 (0.84, 1.04) | 0.93 (0.85, 1.03) | 0.83 (0.73, 0.94) | 0.86 (0.76, 0.97) | 0.004 | 0.96 (0.93, 0.99) |
| Model 4 | 1.00 | 0.93 (0.84, 1.03) | 0.93 (0.85, 1.03) | 0.83 (0.73, 0.94) | 0.87 (0.77, 0.98) | 0.007 | 0.96 (0.93, 0.99) |
| Model 5 | 1.00 | 0.93 (0.84, 1.03) | 0.94 (0.85, 1.04) | 0.83 (0.73, 0.94) | 0.87 (0.77, 0.99) | 0.009 | 0.96 (0.93, 0.99) |
| Model 6 | 1.00 | 0.93 (0.84, 1.03) | 0.93 (0.85, 1.03) | 0.83 (0.73, 0.94) | 0.86 (0.77, 0.98) | 0.006 | 0.96 (0.93, 0.99) |
| Model 7 | 1.00 | 0.93 (0.84, 1.03) | 0.93 (0.85, 1.03) | 0.83 (0.73, 0.94) | 0.86 (0.76, 0.97) | 0.004 | 0.96 (0.93, 0.99) |
| Model 8 | 1.00 | 0.93 (0.84, 1.04) | 0.93 (0.85, 1.03) | 0.83 (0.73, 0.94) | 0.86 (0.77, 0.97) | 0.005 | 0.96 (0.93, 0.99) |
| **Hemorrhagic stroke** |  |  |  |  |  |  |  |
| Basic model | 1.00 | 0.86 (0.79, 0.93) | 0.82 (0.76, 0.88) | 0.77 (0.70, 0.86) | 0.74 (0.67, 0.82) | <0.001 | 0.92 (0.90, 0.95) |
| Model 4 | 1.00 | 0.86 (0.79, 0.93) | 0.83 (0.77, 0.89) | 0.79 (0.71, 0.88) | 0.77 (0.69, 0.85) | <0.001 | 0.93 (0.91, 0.96) |
| Model 5 | 1.00 | 0.86 (0.79, 0.93) | 0.83 (0.77, 0.90) | 0.78 (0.71, 0.87) | 0.75 (0.68, 0.84) | <0.001 | 0.93 (0.90, 0.96) |
| Model 6 | 1.00 | 0.86 (0.79, 0.93) | 0.83 (0.76, 0.89) | 0.78 (0.71, 0.87) | 0.75 (0.68, 0.84) | <0.001 | 0.93 (0.90, 0.96) |
| Model 7 | 1.00 | 0.86 (0.79, 0.93) | 0.82 (0.76, 0.88) | 0.77 (0.70, 0.85) | 0.74 (0.67, 0.83) | <0.001 | 0.92 (0.90, 0.95) |
| Model 8 | 1.00 | 0.86 (0.79, 0.93) | 0.82 (0.76, 0.89) | 0.78 (0.70, 0.86) | 0.75 (0.67, 0.83) | <0.001 | 0.92 (0.90, 0.95) |
| **Ischemic stroke** |  |  |  |  |  |  |  |
| Basic model | 1.00 | 0.98 (0.94, 1.03) | 0.95 (0.91, 1.00) | 0.95 (0.90, 1.00) | 0.90 (0.85, 0.95) | <0.001 | 0.97 (0.96, 0.98) |
| Model 4 | 1.00 | 0.98 (0.94, 1.03) | 0.96 (0.92, 1.00) | 0.96 (0.90, 1.01) | 0.91 (0.86, 0.96) | <0.001 | 0.97 (0.96, 0.99) |
| Model 5 | 1.00 | 0.98 (0.94, 1.03) | 0.96 (0.92, 1.00) | 0.95 (0.90, 1.00) | 0.90 (0.85, 0.95) | <0.001 | 0.97 (0.96, 0.98) |
| Model 6 | 1.00 | 0.98 (0.94, 1.03) | 0.96 (0.92, 1.00) | 0.95 (0.90, 1.01) | 0.90 (0.85, 0.95) | <0.001 | 0.97 (0.96, 0.98) |
| Model 7 | 1.00 | 0.98 (0.94, 1.03) | 0.95 (0.91, 1.00) | 0.95 (0.90, 1.00) | 0.90 (0.85, 0.95) | <0.001 | 0.97 (0.96, 0.98) |
| Model 8 | 1.00 | 0.98 (0.94, 1.03) | 0.96 (0.92, 1.00) | 0.95 (0.90, 1.01) | 0.90 (0.85, 0.95) | <0.001 | 0.97 (0.96, 0.98) |

CVD: cardiovascular disease; IHD: ischemic heart disease; MCE: major coronary events.

Stratified Cox proportional models were used with stratification on survey site and birth cohort (in 5-year intervals). Basic models were adjusted for age (continuous) and sex (men or women), education level (no formal school, primary school, middle school, high school, college, or university or higher), household income (<2,500, 2,500-4,999, 5,000-9,999, 10,000-19,999, 20,000-34,999, or ≥35,000 yuan/year), marital status (married, widowed, divorced or separated, or never married), alcohol consumption (not weekly; ex-regular; not daily; daily consuming 1-15, 15-29, 30-59, or ≥60 g), tobacco smoking (never or occasional; former; current smoking with 1-14, 15-24, or ≥25 cigarettes/day), physical activity in MET-hours/day (continuous), BMI (continuous), waist to hip ratio (continuous), prevalent hypertension (presence or absence), use of aspirin (presence, absence, or unknown), family history of CVD (presence or absence), intake of multivitamin supplementation (presence or absence) and dietary pattern (new affluence, traditional northern, or traditional southern). Model 4 was additionally adjusted for daily energy intake. Model 5 was additionally adjusted for the frequency levels of the other 11 food groups (including rice, wheat products, other staple foods, meat, poultry, fish, dairy products, fresh vegetables, preserved vegetables, fresh fruit and soybean products, with midpoint value assigned to each frequency level and treated as continuous variables). Model 6 adjusted for the dietary pattern without incorporating egg consumption, instead of the dietary pattern incorporating egg consumption. Model 7 adjusted for simultaneous use of aspirin and statins instead of use of aspirin. Model 8 adjusted for a three level of prevalent hypertension (absence, presence with antihypertensive medication, or presence without antihypertensive medication) instead of a two level of prevalent hypertension.

* Tests for linear trend were conducted by assigning 0, 0.5, 2.0, 5.0, 7.0 to the frequency levels from the lowest to the highest and treating the variable as a continuous variable in the Cox models.

† HRs for each 1 egg increment per week were calculated by using the usual amount in the multivariate Cox models.

**Supplemental Table S5 Associations of egg consumption with mortality from cardiovascular disease after further adjustment.**

| **Endpoints** | **Egg consumption** | | | | | **P for linear trend*** | **HR for 1 egg/week**† |
| --- | --- | --- | --- | --- | --- | --- | --- |
|  | **Never/rarely** | **1-3 days/month** | **1-3 d/week** | **4-6 d/week** | **7 d/week** |  |  |
| **CVD** |  |  |  |  |  |  |  |
| Basic model | 1.00 | 0.91 (0.85, 0.98) | 0.88 (0.82, 0.94) | 0.79 (0.73, 0.87) | 0.82 (0.75, 0.89) | <0.001 | 0.94 (0.92, 0.97) |
| Model 4 | 1.00 | 0.91 (0.85, 0.97) | 0.89 (0.83, 0.95) | 0.82 (0.75, 0.89) | 0.85 (0.78, 0.92) | <0.001 | 0.95 (0.93, 0.98) |
| Model 5 | 1.00 | 0.91 (0.85, 0.97) | 0.89 (0.84, 0.96) | 0.81 (0.74, 0.89) | 0.83 (0.77, 0.91) | <0.001 | 0.95 (0.93, 0.97) |
| Model 6 | 1.00 | 0.91 (0.85, 0.97) | 0.89 (0.83, 0.95) | 0.81 (0.74, 0.88) | 0.83 (0.77, 0.91) | <0.001 | 0.95 (0.93, 0.97) |
| Model 7 | 1.00 | 0.91 (0.85, 0.98) | 0.88 (0.82, 0.94) | 0.79 (0.73, 0.87) | 0.82 (0.75, 0.89) | <0.001 | 0.94 (0.92, 0.97) |
| Model 8 | 1.00 | 0.91 (0.85, 0.98) | 0.88 (0.82, 0.94) | 0.80 (0.73, 0.87) | 0.82 (0.75, 0.89) | <0.001 | 0.94 (0.92, 0.97) |
| **IHD** |  |  |  |  |  |  |  |
| Basic model | 1.00 | 0.90 (0.79, 1.02) | 0.94 (0.83, 1.05) | 0.85 (0.73, 0.99) | 0.88 (0.77, 1.02) | 0.131 | 0.97 (0.93, 1.01) |
| Model 4 | 1.00 | 0.90 (0.79, 1.02) | 0.94 (0.84, 1.06) | 0.87 (0.75, 1.02) | 0.91 (0.79, 1.06) | 0.337 | 0.98 (0.94, 1.02) |
| Model 5 | 1.00 | 0.89 (0.79, 1.02) | 0.95 (0.85, 1.07) | 0.87 (0.74, 1.01) | 0.91 (0.78, 1.05) | 0.280 | 0.98 (0.94, 1.02) |
| Model 6 | 1.00 | 0.90 (0.79, 1.02) | 0.94 (0.84, 1.06) | 0.86 (0.74, 1.00) | 0.90 (0.78, 1.04) | 0.227 | 0.98 (0.94, 1.02) |
| Model 7 | 1.00 | 0.90 (0.79, 1.02) | 0.94 (0.83, 1.05) | 0.85 (0.73, 0.99) | 0.88 (0.77, 1.02) | 0.132 | 0.97 (0.93, 1.01) |
| Model 8 | 1.00 | 0.90 (0.79, 1.02) | 0.94 (0.83, 1.05) | 0.85 (0.73, 0.99) | 0.89 (0.77, 1.02) | 0.140 | 0.97 (0.94, 1.01) |
| **Hemorrhagic stroke** |  |  |  |  |  |  |  |
| Basic model | 1.00 | 0.87 (0.78, 0.97) | 0.79 (0.71, 0.88) | 0.74 (0.65, 0.86) | 0.72 (0.62, 0.84) | <0.001 | 0.91 (0.87, 0.95) |
| Model 4 | 1.00 | 0.86 (0.77, 0.97) | 0.80 (0.72, 0.89) | 0.77 (0.66, 0.88) | 0.75 (0.65, 0.87) | 0.001 | 0.92 (0.88, 0.96) |
| Model 5 | 1.00 | 0.87 (0.78, 0.97) | 0.81 (0.73, 0.90) | 0.77 (0.66, 0.88) | 0.74 (0.64, 0.86) | <0.001 | 0.92 (0.88, 0.96) |
| Model 6 | 1.00 | 0.86 (0.77, 0.97) | 0.80 (0.72, 0.89) | 0.75 (0.65, 0.87) | 0.73 (0.63, 0.85) | <0.001 | 0.91 (0.87, 0.95) |
| Model 7 | 1.00 | 0.87 (0.78, 0.97) | 0.79 (0.71, 0.88) | 0.74 (0.65, 0.86) | 0.72 (0.62, 0.84) | <0.001 | 0.91 (0.87, 0.95) |
| Model 8 | 1.00 | 0.87 (0.78, 0.97) | 0.79 (0.71, 0.88) | 0.75 (0.65, 0.86) | 0.73 (0.63, 0.84) | <0.001 | 0.91 (0.87, 0.95) |
| **Ischemic stroke** |  |  |  |  |  |  |  |
| Basic model | 1.00 | 1.05 (0.83, 1.31) | 1.02 (0.82, 1.27) | 1.00 (0.74, 1.33) | 0.93 (0.71, 1.22) | 0.388 | 0.97 (0.90, 1.04) |
| Model 4 | 1.00 | 1.05 (0.83, 1.32) | 1.05 (0.85, 1.31) | 1.05 (0.78, 1.41) | 1.00 (0.75, 1.32) | 0.816 | 0.99 (0.92, 1.07) |
| Model 5 | 1.00 | 1.04 (0.82, 1.30) | 1.05 (0.84, 1.30) | 1.01 (0.75, 1.35) | 0.95 (0.72, 1.26) | 0.526 | 0.98 (0.91, 1.05) |
| Model 6 | 1.00 | 1.05 (0.83, 1.32) | 1.05 (0.84, 1.31) | 1.06 (0.79, 1.42) | 1.02 (0.77, 1.35) | 0.979 | 1.00 (0.93, 1.08) |
| Model 7 | 1.00 | 1.05 (0.83, 1.31) | 1.02 (0.82, 1.27) | 1.00 (0.74, 1.33) | 0.93 (0.70, 1.22) | 0.388 | 0.97 (0.90, 1.04) |
| Model 8 | 1.00 | 1.05 (0.83, 1.32) | 1.03 (0.83, 1.28) | 1.00 (0.75, 1.34) | 0.93 (0.71, 1.23) | 0.412 | 0.97 (0.90, 1.05) |

CVD: cardiovascular disease; IHD: ischemic heart disease.

Stratified Cox proportional models were used with stratification on survey site and birth cohort (in 5-year intervals). Basic models were adjusted for age (continuous) and sex (men or women), education level (no formal school, primary school, middle school, high school, college, or university or higher), household income (<2,500, 2,500-4,999, 5,000-9,999, 10,000-19,999, 20,000-34,999, or ≥35,000 yuan/year), marital status (married, widowed, divorced or separated, or never married), alcohol consumption (not weekly; ex-regular; not daily; daily consuming 1-15, 15-29, 30-59, or ≥60 g), tobacco smoking (never or occasional; former; current smoking with 1-14, 15-24, or ≥25 cigarettes/day), physical activity in MET-hours/day (continuous), BMI (continuous), waist to hip ratio (continuous), prevalent hypertension (presence or absence), use of aspirin (presence, absence, or unknown), family history of CVD (presence or absence), intake of multivitamin supplementation (presence or absence) and dietary pattern (new affluence, traditional northern, or traditional southern). . Model 4 was additionally adjusted for daily energy intake. Model 5 was additionally adjusted for the frequency levels of the other 11 food groups (including rice, wheat products, other staple foods, meat, poultry, fish, dairy products, fresh vegetables, preserved vegetables, fresh fruit and soybean products, with midpoint value assigned to each frequency level and treated as continuous variables). Model 6 adjusted for the dietary pattern without incorporating egg consumption, instead of the dietary pattern incorporating egg consumption. Model 7 adjusted for simultaneous use of aspirin and statins instead of use of aspirin. Model 8 adjusted for a three level of prevalent hypertension (absence, presence with antihypertensive medication, or presence without antihypertensive medication) instead of a two level of prevalent hypertension.

* Tests for interaction were performed using likelihood ratio tests, which involved comparing models with and without interaction terms between the strata variable and egg consumption (as a multinomial variable).

† HRs for each 1 egg increment per week were calculated by using the usual amount in the multivariate Cox models

**Supplemental Table S6. Subgroup analysis of associations between egg consumption and risk of cardiovascular disease according to potential baseline risk factors.**

| **Subgroups** | **Egg consumption** | | | | | | | | | | | | | | |
| --- | --- | --- | --- | --- | --- | --- | --- | --- | --- | --- | --- | --- | --- | --- | --- |
|  | **Never/rarely** | |  | **1-3 days/month** | |  | **1-3 d/week** | |  | **4-6 d/week** | |  | **7 d/week** | | ***P* for interaction†** |
|  | **No.*** | **HR** |  | **No.*** | **HR(%95CI)** |  | **No.*** | **HR(%95CI)** |  | **No.*** | **HR(%95CI)** |  | **No.*** | **HR(%95CI)** |  |
| **Sex** |  |  |  |  |  |  |  |  |  |  |  |  |  |  |  |
| Women | 5,245 | 1.00 |  | 10,042 | 0.97 (0.94, 1.01) |  | 20,952 | 0.92 (0.89, 0.95) |  | 4,708 | 0.90 (0.86, 0.94) |  | 6,558 | 0.90 (0.87, 0.94) | 0.005 |
| Men | 2,880 | 1.00 |  | 7,044 | 0.97 (0.93, 1.02) |  | 17,195 | 0.92 (0.88, 0.96) |  | 3,872 | 0.90 (0.85, 0.94) |  | 5,481 | 0.88 (0.84, 0.92) |  |
| **Age (yr)** |  |  |  |  |  |  |  |  |  |  |  |  |  |  |  |
| <50 | 1,591 | 1.00 |  | 4,048 | 1.00 (0.95, 1.06) |  | 10,303 | 0.95 (0.90, 1.00) |  | 2,569 | 0.95 (0.89, 1.02) |  | 2,956 | 0.93 (0.87, 1.00) | 0.213 |
| 50-60 | 2,658 | 1.00 |  | 5,672 | 1.00 (0.96, 1.05) |  | 12,554 | 0.94 (0.90, 0.98) |  | 2,808 | 0.90 (0.85, 0.95) |  | 3,756 | 0.90 (0.85, 0.95) |  |
| ≥60 | 3,876 | 1.00 |  | 7,366 | 0.94 (0.91, 0.98) |  | 15,290 | 0.90 (0.87, 0.93) |  | 3,203 | 0.89 (0.84, 0.93) |  | 5,327 | 0.86 (0.82, 0.90) |  |
| **Area** |  |  |  |  |  |  |  |  |  |  |  |  |  |  |  |
| Urban | 2,655 | 1.00 |  | 5,140 | 0.94 (0.90, 0.99) |  | 16,643 | 0.89 (0.85, 0.93) |  | 2,820 | 0.86 (0.81, 0.91) |  | 7,614 | 0.86 (0.82, 0.91) | 0.625 |
| Rural | 5,470 | 1.00 |  | 11,946 | 0.98 (0.95, 1.01) |  | 21,504 | 0.93 (0.90, 0.96) |  | 5,760 | 0.91 (0.87, 0.95) |  | 4,425 | 0.91 (0.87, 0.95) |  |
| **Highest education level** |  |  |  |  |  |  |  |  |  |  |  |  |  |  |  |
| Primary school and below | 5,839 | 1.00 |  | 12,059 | 0.97 (0.94, 1.00) |  | 21,588 | 0.92 (0.89, 0.95) |  | 4,821 | 0.89 (0.86, 0.93) |  | 4,717 | 0.90 (0.86, 0.93) | 0.810 |
| Middle or high school | 2,115 | 1.00 |  | 4,587 | 0.97 (0.92, 1.02) |  | 14,220 | 0.91 (0.87, 0.96) |  | 3,234 | 0.91 (0.86, 0.96) |  | 5,865 | 0.89 (0.84, 0.94) |  |
| College and above | 171 | 1.00 |  | 440 | 1.05 (0.88, 1.26) |  | 2,339 | 1.01 (0.86, 1.18) |  | 525 | 0.94 (0.79, 1.13) |  | 1,457 | 0.93 (0.79, 1.10) |  |
| **Household income (yuan/yr)** |  |  |  |  |  |  |  |  |  |  |  |  |  |  |  |
| <10,000 | 4,228 | 1.00 |  | 6,754 | 0.96 (0.92, 0.99) |  | 10,045 | 0.92 (0.88, 0.95) |  | 2,029 | 0.87 (0.83, 0.92) |  | 2,789 | 0.88 (0.83, 0.92) | 0.159 |
| 10,000-19,999 | 2,303 | 1.00 |  | 4,417 | 0.94 (0.89, 0.99) |  | 11,693 | 0.92 (0.87, 0.96) |  | 2,570 | 0.90 (0.85, 0.96) |  | 3,803 | 0.89 (0.84, 0.94) |  |
| ≥20,000 | 1,594 | 1.00 |  | 5,915 | 1.04 (0.99, 1.10) |  | 16,409 | 0.96 (0.91, 1.01) |  | 3,981 | 0.94 (0.89, 1.00) |  | 5,447 | 0.92 (0.87, 0.98) |  |
| **Alcohol consumption** |  |  |  |  |  |  |  |  |  |  |  |  |  |  |  |
| Not current | 7,252 | 1.00 |  | 15,047 | 0.97 (0.94, 1.00) |  | 32,187 | 0.92 (0.90, 0.95) |  | 7,360 | 0.90 (0.87, 0.93) |  | 9,728 | 0.90 (0.87, 0.93) | 0.737 |
| Current | 873 | 1.00 |  | 2,039 | 0.98 (0.90, 1.06) |  | 5,960 | 0.91 (0.85, 0.99) |  | 1,220 | 0.90 (0.82, 0.99) |  | 2,311 | 0.88 (0.80, 0.95) |  |
| **Tobacco smoking** |  |  |  |  |  |  |  |  |  |  |  |  |  |  |  |
| Not current | 5,754 | 1.00 |  | 11,704 | 0.96 (0.93, 0.99) |  | 25,810 | 0.91 (0.88, 0.94) |  | 5,769 | 0.89 (0.86, 0.93) |  | 8,140 | 0.89 (0.86, 0.93) | 0.150 |
| Current | 2,371 | 1.00 |  | 5,382 | 1.00 (0.95, 1.05) |  | 12,337 | 0.94 (0.90, 0.99) |  | 2,811 | 0.91 (0.86, 0.97) |  | 3,899 | 0.89 (0.84, 0.94) |  |
| **Physical activity (MET-hr/day)** |  |  |  |  |  |  |  |  |  |  |  |  |  |  |  |
| Low | 3,935 | 1.00 |  | 7,205 | 0.97 (0.93, 1.01) |  | 17,987 | 0.93 (0.90, 0.96) |  | 3,768 | 0.90 (0.86, 0.95) |  | 6,472 | 0.89 (0.86, 0.94) | 0.101 |
| Middle | 2,488 | 1.00 |  | 5,275 | 0.99 (0.94, 1.04) |  | 11,051 | 0.92 (0.87, 0.96) |  | 2,728 | 0.92 (0.87, 0.97) |  | 3,577 | 0.91 (0.86, 0.96) |  |
| High | 1,702 | 1.00 |  | 4,606 | 0.96 (0.90, 1.01) |  | 9,109 | 0.92 (0.87, 0.97) |  | 2,084 | 0.88 (0.82, 0.94) |  | 1,990 | 0.88 (0.82, 0.94) |  |
| **Dietary pattern** |  |  |  |  |  |  |  |  |  |  |  |  |  |  |  |
| New affluence | 555 | 1.00 |  | 1,105 | 0.99 (0.89, 1.09) |  | 7,935 | 0.93 (0.85, 1.01) |  | 2,812 | 0.89 (0.81, 0.97) |  | 7,919 | 0.87 (0.80, 0.95) | 0.031 |
| Traditional southern | 4,710 | 1.00 |  | 10,562 | 0.94 (0.91, 0.97) |  | 21,602 | 0.89 (0.86, 0.92) |  | 3,723 | 0.88 (0.84, 0.93) |  | 1,243 | 0.85 (0.80, 0.90) |  |
| Traditional northern | 2,860 | 1.00 |  | 5,419 | 1.01 (0.96, 1.06) |  | 8,610 | 0.98 (0.93, 1.02) |  | 2,045 | 0.94 (0.89, 1.00) |  | 2,877 | 0.97 (0.92, 1.02) |  |
| **BMI (kg/m^2^)** |  |  |  |  |  |  |  |  |  |  |  |  |  |  |  |
| <24.0 | 4,453 | 1.00 |  | 9,967 | 0.97 (0.94, 1.01) |  | 20,589 | 0.92 (0.89, 0.95) |  | 4,960 | 0.91 (0.87, 0.95) |  | 5,498 | 0.89 (0.85, 0.92) | 0.005 |
| 24.0-28.0 | 2,616 | 1.00 |  | 5,339 | 0.97 (0.92, 1.01) |  | 12,921 | 0.90 (0.87, 0.95) |  | 2,728 | 0.89 (0.84, 0.94) |  | 4,558 | 0.89 (0.85, 0.94) |  |
| ≥28.0 | 1,056 | 1.00 |  | 1,780 | 0.99 (0.91, 1.07) |  | 4,637 | 1.00 (0.93, 1.07) |  | 892 | 0.90 (0.82, 0.99) |  | 1,983 | 0.95 (0.87, 1.04) |  |
| **Hypertension** |  |  |  |  |  |  |  |  |  |  |  |  |  |  |  |
| Yes | 3,656 | 1.00 |  | 8,151 | 0.99 (0.95, 1.03) |  | 18,879 | 0.92 (0.89, 0.95) |  | 4,618 | 0.91 (0.87, 0.96) |  | 6,355 | 0.90 (0.86, 0.95) | 0.059 |
| No | 4,469 | 1.00 |  | 8,935 | 0.96 (0.93, 1.00) |  | 19,268 | 0.93 (0.90, 0.96) |  | 3,962 | 0.89 (0.85, 0.93) |  | 5,684 | 0.89 (0.86, 0.93) |  |

HR indicates hazard ratios; CI confidence interval.

Stratified Cox proportional models were used with stratification on survey site and birth cohort (in 5-year intervals), except for subgroup and interaction analyses according to age at recruitment (only with stratification on survey site). Multivariate models were adjusted for age at recruitment (continuous) and sex (men or women); model 2: additionally included education level (no formal school, primary school, middle school, high school, college, or university or higher), household income (<2,500, 2,500-4,999, 5,000-9,999, 10,000-19,999, 20,000-34,999, or ≥35,000 yuan/year), marital status (married, widowed, divorced or separated, or never married), alcohol consumption (not weekly; ex-regular; not daily; daily consuming 1-15, 15-29, 30-59, or ≥60 g), tobacco smoking (never or occasional; former; current smoking with 1-14, 15-24, or ≥25 cigarettes/day), physical activity in MET-hours/day (continuous), BMI (continuous), waist to hip ratio (continuous), prevalent hypertension (presence or absence), use of aspirin (presence, absence, or unknown), family history of CVD (presence or absence); model 3: additionally included intake of multivitamin supplementation (presence or absence) and dietary pattern (new affluence, traditional northern, or traditional southern), as appropriate.

* No. of incident cardiovascular disease cases.

† Tests for interaction were performed using likelihood ratio tests, which involved comparing models with and without interaction terms between the strata variable and egg consumption (as a multinomial variable).

**Supplemental Table S7. Subgroup analysis of associations between egg consumption and risk of ischemic heart disease according to potential baseline risk factors.**

| **Subgroups** | **Egg consumption** | | | | | | | | | | | | | | |
| --- | --- | --- | --- | --- | --- | --- | --- | --- | --- | --- | --- | --- | --- | --- | --- |
|  | **Never/rarely** | |  | **1-3 days/month** | |  | **1-3 d/week** | |  | **4-6 d/week** | |  | **7 d/week** | | ***P* for interaction†** |
|  | **No.*** | **HR** |  | **No.*** | **HR(%95CI)** |  | **No.*** | **HR(%95CI)** |  | **No.*** | **HR(%95CI)** |  | **No.*** | **HR(%95CI)** |  |
| **Sex** |  |  |  |  |  |  |  |  |  |  |  |  |  |  |  |
| Women | 1,908 | 1.00 |  | 3,316 | 0.96 (0.90, 1.01) |  | 7,738 | 0.93 (0.89, 0.98) |  | 1,689 | 0.86 (0.80, 0.92) |  | 2,927 | 0.87 (0.82, 0.93) | 0.346 |
| Men | 958 | 1.00 |  | 2,213 | 0.94 (0.87, 1.02) |  | 5,803 | 0.91 (0.84, 0.97) |  | 1,380 | 0.86 (0.79, 0.94) |  | 2,237 | 0.89 (0.82, 0.97) |  |
| **Age (yr)** |  |  |  |  |  |  |  |  |  |  |  |  |  |  |  |
| <50 | 483 | 1.00 |  | 1,148 | 1.01 (0.91, 1.12) |  | 3,161 | 0.96 (0.87, 1.06) |  | 757 | 0.89 (0.79, 1.00) |  | 1,068 | 0.90 (0.81, 1.01) | 0.743 |
| 50-60 | 923 | 1.00 |  | 1,795 | 0.99 (0.91, 1.07) |  | 4,324 | 0.93 (0.87, 1.01) |  | 966 | 0.85 (0.77, 0.93) |  | 1,632 | 0.89 (0.82, 0.98) |  |
| ≥60 | 1,460 | 1.00 |  | 2,586 | 0.92 (0.86, 0.98) |  | 6,056 | 0.91 (0.85, 0.96) |  | 1,346 | 0.88 (0.81, 0.95) |  | 2,464 | 0.86 (0.80, 0.93) |  |
| **Area** |  |  |  |  |  |  |  |  |  |  |  |  |  |  |  |
| Urban | 997 | 1.00 |  | 1,741 | 0.88 (0.81, 0.95) |  | 6,558 | 0.83 (0.77, 0.89) |  | 1,137 | 0.75 (0.68, 0.82) |  | 3,582 | 0.79 (0.73, 0.85) | 0.014 |
| Rural | 1,869 | 1.00 |  | 3,788 | 0.98 (0.93, 1.04) |  | 6,983 | 0.96 (0.91, 1.02) |  | 1,932 | 0.92 (0.86, 0.98) |  | 1,582 | 0.93 (0.87, 1.00) |  |
| **Highest education level** |  |  |  |  |  |  |  |  |  |  |  |  |  |  |  |
| Primary school and below | 1,987 | 1.00 |  | 3,780 | 0.96 (0.91, 1.01) |  | 7,353 | 0.96 (0.91, 1.01) |  | 1,686 | 0.89 (0.83, 0.95) |  | 1,945 | 0.90 (0.84, 0.97) | 0.011 |
| Middle or high school | 795 | 1.00 |  | 1,546 | 0.91 (0.84, 0.99) |  | 5,176 | 0.85 (0.79, 0.92) |  | 1,168 | 0.81 (0.74, 0.89) |  | 2,530 | 0.83 (0.76, 0.91) |  |
| College and above | 84 | 1.00 |  | 203 | 0.98 (0.76, 1.27) |  | 1,012 | 0.80 (0.64, 1.01) |  | 215 | 0.70 (0.54, 0.91) |  | 689 | 0.75 (0.59, 0.95) |  |
| **Household income (yuan/yr)** |  |  |  |  |  |  |  |  |  |  |  |  |  |  |  |
| <10,000 | 1,485 | 1.00 |  | 2,371 | 0.95 (0.89, 1.02) |  | 3,607 | 0.91 (0.85, 0.96) |  | 765 | 0.87 (0.79, 0.95) |  | 1,103 | 0.87 (0.80, 0.95) | 0.592 |
| 10,000-19,999 | 804 | 1.00 |  | 1,420 | 0.90 (0.83, 0.98) |  | 4,353 | 0.91 (0.84, 0.99) |  | 924 | 0.83 (0.75, 0.92) |  | 1,622 | 0.86 (0.78, 0.94) |  |
| ≥20,000 | 577 | 1.00 |  | 1,738 | 0.99 (0.90, 1.09) |  | 5,581 | 0.94 (0.86, 1.03) |  | 1,380 | 0.89 (0.80, 0.98) |  | 2,439 | 0.91 (0.82, 1.00) |  |
| **Alcohol consumption** |  |  |  |  |  |  |  |  |  |  |  |  |  |  |  |
| Not current | 2,587 | 1.00 |  | 4,952 | 0.96 (0.91, 1.00) |  | 11,571 | 0.92 (0.88, 0.97) |  | 2,640 | 0.86 (0.81, 0.91) |  | 4,228 | 0.88 (0.84, 0.93) | 0.793 |
| Current | 279 | 1.00 |  | 577 | 0.91 (0.78, 1.05) |  | 1,970 | 0.90 (0.79, 1.02) |  | 429 | 0.86 (0.73, 1.01) |  | 936 | 0.86 (0.75, 1.00) |  |
| **Tobacco smoking** |  |  |  |  |  |  |  |  |  |  |  |  |  |  |  |
| Not current | 2,048 | 1.00 |  | 3,754 | 0.93 (0.88, 0.98) |  | 9,335 | 0.92 (0.87, 0.97) |  | 2,056 | 0.85 (0.80, 0.91) |  | 3,547 | 0.87 (0.82, 0.92) | 0.155 |
| Current | 818 | 1.00 |  | 1,775 | 1.00 (0.92, 1.09) |  | 4,206 | 0.93 (0.86, 1.01) |  | 1,013 | 0.88 (0.80, 0.97) |  | 1,617 | 0.92 (0.84, 1.01) |  |
| **Physical activity (MET-hr/day)** |  |  |  |  |  |  |  |  |  |  |  |  |  |  |  |
| Low | 1,495 | 1.00 |  | 2,542 | 0.91 (0.86, 0.98) |  | 7,114 | 0.91 (0.86, 0.96) |  | 1,462 | 0.83 (0.77, 0.90) |  | 2,971 | 0.87 (0.81, 0.93) | 0.116 |
| Middle | 825 | 1.00 |  | 1,660 | 0.99 (0.91, 1.08) |  | 3,771 | 0.93 (0.86, 1.01) |  | 951 | 0.92 (0.83, 1.01) |  | 1,493 | 0.92 (0.83, 1.01) |  |
| High | 546 | 1.00 |  | 1,327 | 0.98 (0.89, 1.09) |  | 2,656 | 0.95 (0.86, 1.04) |  | 656 | 0.85 (0.76, 0.96) |  | 700 | 0.85 (0.75, 0.96) |  |
| **Dietary pattern** |  |  |  |  |  |  |  |  |  |  |  |  |  |  |  |
| New affluence | 290 | 1.00 |  | 483 | 0.85 (0.73, 0.98) |  | 3,416 | 0.75 (0.67, 0.85) |  | 1,075 | 0.66 (0.58, 0.75) |  | 3,660 | 0.71 (0.63, 0.80) | <0.001 |
| Traditional southern | 1,522 | 1.00 |  | 3,021 | 0.92 (0.86, 0.98) |  | 6,891 | 0.91 (0.85, 0.96) |  | 1,219 | 0.88 (0.81, 0.96) |  | 404 | 0.81 (0.73, 0.91) |  |
| Traditional northern | 1,054 | 1.00 |  | 2,025 | 1.02 (0.95, 1.10) |  | 3,234 | 0.99 (0.92, 1.06) |  | 775 | 0.95 (0.87, 1.05) |  | 1,100 | 0.98 (0.90, 1.07) |  |
| **BMI (kg/m^2^)** |  |  |  |  |  |  |  |  |  |  |  |  |  |  |  |
| <24.0 | 1,447 | 1.00 |  | 3,106 | 0.98 (0.92, 1.05) |  | 6,894 | 0.95 (0.90, 1.01) |  | 1,667 | 0.88 (0.82, 0.95) |  | 2,139 | 0.89 (0.83, 0.95) | 0.046 |
| 24.0-28.0 | 986 | 1.00 |  | 1,764 | 0.90 (0.84, 0.98) |  | 4,739 | 0.87 (0.81, 0.93) |  | 1,050 | 0.85 (0.78, 0.93) |  | 2,003 | 0.84 (0.78, 0.92) |  |
| ≥28.0 | 433 | 1.00 |  | 659 | 0.95 (0.84, 1.07) |  | 1,908 | 0.98 (0.88, 1.09) |  | 352 | 0.81 (0.70, 0.95) |  | 1,022 | 0.97 (0.85, 1.10) |  |
| **Hypertension** |  |  |  |  |  |  |  |  |  |  |  |  |  |  |  |
| Yes | 1,297 | 1.00 |  | 2,617 | 0.95 (0.89, 1.02) |  | 6,613 | 0.90 (0.84, 0.96) |  | 1,620 | 0.85 (0.78, 0.91) |  | 2,632 | 0.84 (0.79, 0.91) | 0.085 |
| No | 1,569 | 1.00 |  | 2,912 | 0.95 (0.89, 1.01) |  | 6,928 | 0.94 (0.89, 1.00) |  | 1,449 | 0.87 (0.80, 0.94) |  | 2,532 | 0.92 (0.86, 0.99) |  |

HR indicates hazard ratios; CI confidence interval.

Stratified Cox proportional models were used with stratification on survey site and birth cohort (in 5-year intervals), except for subgroup and interaction analyses according to age at recruitment (only with stratification on survey site). Multivariate models were adjusted for age at recruitment (continuous) and sex (men or women); model 2: additionally included education level (no formal school, primary school, middle school, high school, college, or university or higher), household income (<2,500, 2,500-4,999, 5,000-9,999, 10,000-19,999, 20,000-34,999, or ≥35,000 yuan/year), marital status (married, widowed, divorced or separated, or never married), alcohol consumption (not weekly; ex-regular; not daily; daily consuming 1-15, 15-29, 30-59, or ≥60 g), tobacco smoking (never or occasional; former; current smoking with 1-14, 15-24, or ≥25 cigarettes/day), physical activity in MET-hours/day (continuous), BMI (continuous), waist to hip ratio (continuous), prevalent hypertension (presence or absence), use of aspirin (presence, absence, or unknown), family history of CVD (presence or absence); model 3: additionally included intake of multivitamin supplementation (presence or absence) and dietary pattern (new affluence, traditional northern, or traditional southern), as appropriate.

* No. of incident ischemic heart disease cases.

† The tests for interaction were performed using likelihood ratio tests, which involved comparing models with and without interaction terms between the strata variable and egg consumption (as a multinomial variable).

**Supplemental Table S8. Subgroup analysis of associations between egg consumption and major coronary events according to potential baseline risk factors.**

| **Subgroups** | **Egg consumption** | | | | | | | | | | | | | | |
| --- | --- | --- | --- | --- | --- | --- | --- | --- | --- | --- | --- | --- | --- | --- | --- |
|  | **Never/rarely** | |  | **1-3 days/month** | |  | **1-3 d/week** | |  | **4-6 d/week** | |  | **7 d/week** | | ***P* for interaction†** |
|  | **No.*** | **HR** |  | **No.*** | **HR(%95CI)** |  | **No.*** | **HR(%95CI)** |  | **No.*** | **HR(%95CI)** |  | **No.*** | **HR(%95CI)** |  |
| **Sex** |  |  |  |  |  |  |  |  |  |  |  |  |  |  |  |
| Women | 305 | 1.00 |  | 496 | 0.97 (0.84, 1.12) |  | 845 | 0.91 (0.79, 1.05) |  | 204 | 0.89 (0.74, 1.07) |  | 275 | 0.88 (0.73, 1.06) | 0.019 |
| Men | 260 | 1.00 |  | 537 | 0.91 (0.78, 1.06) |  | 1,374 | 0.95 (0.83, 1.09) |  | 309 | 0.81 (0.68, 0.96) |  | 498 | 0.87 (0.74, 1.03) |  |
| **Age (yr)** |  |  |  |  |  |  |  |  |  |  |  |  |  |  |  |
| <50 | 46 | 1.00 |  | 135 | 1.31 (0.93, 1.83) |  | 287 | 1.20 (0.86, 1.66) |  | 72 | 1.15 (0.78, 1.69) |  | 88 | 1.17 (0.79, 1.72) | 0.724 |
| 50-60 | 129 | 1.00 |  | 224 | 0.86 (0.69, 1.07) |  | 497 | 0.89 (0.72, 1.09) |  | 117 | 0.74 (0.57, 0.96) |  | 158 | 0.82 (0.64, 1.06) |  |
| ≥60 | 390 | 1.00 |  | 674 | 0.91 (0.80, 1.03) |  | 1,435 | 0.92 (0.82, 1.04) |  | 324 | 0.83 (0.71, 0.97) |  | 527 | 0.84 (0.72, 0.97) |  |
| **Area** |  |  |  |  |  |  |  |  |  |  |  |  |  |  |  |
| Urban | 165 | 1.00 |  | 286 | 0.90 (0.75, 1.10) |  | 849 | 1.02 (0.85, 1.22) |  | 140 | 0.97 (0.76, 1.23) |  | 443 | 0.99 (0.81, 1.23) | 0.245 |
| Rural | 400 | 1.00 |  | 747 | 0.94 (0.83, 1.06) |  | 1,370 | 0.90 (0.80, 1.02) |  | 373 | 0.79 (0.68, 0.92) |  | 330 | 0.81 (0.70, 0.95) |  |
| **Highest education level** |  |  |  |  |  |  |  |  |  |  |  |  |  |  |  |
| Primary school and below | 435 | 1.00 |  | 758 | 0.92 (0.82, 1.04) |  | 1,456 | 0.94 (0.84, 1.06) |  | 338 | 0.83 (0.72, 0.97) |  | 396 | 0.87 (0.75, 1.01) | 0.717 |
| Middle or high school | 125 | 1.00 |  | 249 | 0.91 (0.73, 1.13) |  | 656 | 0.85 (0.69, 1.03) |  | 156 | 0.78 (0.61, 1.00) |  | 301 | 0.79 (0.63, 0.99) |  |
| College and above | 5 | 1.00 |  | 26 | 1.76 (0.67, 4.61) |  | 107 | 1.80 (0.72, 4.48) |  | 19 | 1.10 (0.40, 3.06) |  | 76 | 1.79 (0.70, 4.59) |  |
| **Household income (yuan/yr)** |  |  |  |  |  |  |  |  |  |  |  |  |  |  |  |
| <10,000 | 353 | 1.00 |  | 549 | 0.91 (0.79, 1.04) |  | 801 | 0.85 (0.74, 0.97) |  | 172 | 0.79 (0.65, 0.95) |  | 239 | 0.76 (0.64, 0.91) | 0.412 |
| 10,000-19,999 | 138 | 1.00 |  | 243 | 0.91 (0.73, 1.12) |  | 700 | 0.97 (0.80, 1.18) |  | 148 | 0.86 (0.67, 1.09) |  | 261 | 1.04 (0.83, 1.31) |  |
| ≥20,000 | 74 | 1.00 |  | 241 | 1.11 (0.85, 1.45) |  | 718 | 1.14 (0.89, 1.46) |  | 193 | 0.99 (0.75, 1.31) |  | 273 | 0.99 (0.75, 1.31) |  |
| **Alcohol consumption** |  |  |  |  |  |  |  |  |  |  |  |  |  |  |  |
| Not current | 499 | 1.00 |  | 923 | 0.95 (0.85, 1.06) |  | 1,856 | 0.96 (0.86, 1.06) |  | 432 | 0.85 (0.74, 0.97) |  | 605 | 0.89 (0.78, 1.01) | 0.738 |
| Current | 66 | 1.00 |  | 110 | 0.80 (0.58, 1.09) |  | 363 | 0.79 (0.60, 1.04) |  | 81 | 0.72 (0.51, 1.02) |  | 168 | 0.74 (0.54, 1.01) |  |
| **Tobacco smoking** |  |  |  |  |  |  |  |  |  |  |  |  |  |  |  |
| Not current | 349 | 1.00 |  | 603 | 0.92 (0.81, 1.06) |  | 1,216 | 0.94 (0.83, 1.07) |  | 261 | 0.81 (0.68, 0.96) |  | 400 | 0.87 (0.74, 1.02) | 0.354 |
| Current | 216 | 1.00 |  | 430 | 0.96 (0.81, 1.13) |  | 1,003 | 0.94 (0.80, 1.10) |  | 252 | 0.87 (0.72, 1.06) |  | 373 | 0.88 (0.74, 1.06) |  |
| **Physical activity (MET-hr/day)** |  |  |  |  |  |  |  |  |  |  |  |  |  |  |  |
| Low | 353 | 1.00 |  | 594 | 0.91 (0.79, 1.04) |  | 1,410 | 0.91 (0.81, 1.03) |  | 284 | 0.77 (0.65, 0.91) |  | 493 | 0.78 (0.67, 0.91) | 0.653 |
| Middle | 133 | 1.00 |  | 265 | 0.98 (0.79, 1.21) |  | 487 | 1.00 (0.81, 1.22) |  | 144 | 1.00 (0.77, 1.28) |  | 174 | 1.01 (0.79, 1.30) |  |
| High | 79 | 1.00 |  | 174 | 0.98 (0.75, 1.29) |  | 322 | 0.93 (0.72, 1.21) |  | 85 | 0.81 (0.59, 1.12) |  | 106 | 1.00 (0.73, 1.37) |  |
| **Dietary pattern** |  |  |  |  |  |  |  |  |  |  |  |  |  |  |  |
| New affluence | 36 | 1.00 |  | 65 | 0.84 (0.56, 1.27) |  | 380 | 0.77 (0.55, 1.09) |  | 133 | 0.66 (0.45, 0.95) |  | 473 | 0.78 (0.55, 1.11) | 0.706 |
| Traditional southern | 289 | 1.00 |  | 535 | 0.93 (0.80, 1.08) |  | 1,210 | 0.95 (0.82, 1.09) |  | 228 | 0.83 (0.69, 1.01) |  | 75 | 0.75 (0.58, 0.98) |  |
| Traditional northern | 240 | 1.00 |  | 433 | 0.95 (0.81, 1.11) |  | 629 | 0.95 (0.81, 1.10) |  | 152 | 0.91 (0.74, 1.12) |  | 225 | 0.91 (0.75, 1.10) |  |
| **BMI (kg/m^2^)** |  |  |  |  |  |  |  |  |  |  |  |  |  |  |  |
| <24.0 | 328 | 1.00 |  | 638 | 0.96 (0.84, 1.10) |  | 1,303 | 0.98 (0.86, 1.11) |  | 310 | 0.82 (0.69, 0.97) |  | 368 | 0.85 (0.72, 1.00) | 0.871 |
| 24.0-28.0 | 166 | 1.00 |  | 293 | 0.91 (0.75, 1.10) |  | 683 | 0.90 (0.75, 1.07) |  | 153 | 0.87 (0.69, 1.09) |  | 277 | 0.89 (0.72, 1.10) |  |
| ≥28.0 | 71 | 1.00 |  | 102 | 0.91 (0.67, 1.23) |  | 233 | 0.86 (0.65, 1.14) |  | 50 | 0.82 (0.55, 1.20) |  | 128 | 0.90 (0.64, 1.26) |  |
| **Hypertension** |  |  |  |  |  |  |  |  |  |  |  |  |  |  |  |
| Yes | 204 | 1.00 |  | 391 | 0.93 (0.78, 1.10) |  | 830 | 0.90 (0.76, 1.06) |  | 221 | 0.87 (0.71, 1.06) |  | 307 | 0.85 (0.70, 1.04) | 0.629 |
| No | 361 | 1.00 |  | 642 | 0.93 (0.82, 1.06) |  | 1,389 | 0.96 (0.85, 1.08) |  | 292 | 0.80 (0.68, 0.94) |  | 466 | 0.87 (0.75, 1.01) |  |

HR indicates hazard ratios; CI confidence interval.

Stratified Cox proportional models were used with stratification on survey site and birth cohort (in 5-year intervals), except for subgroup and interaction analyses according to age at recruitment (only with stratification on survey site). Multivariate models were adjusted for age at recruitment (continuous) and sex (men or women); model 2: additionally included education level (no formal school, primary school, middle school, high school, college, or university or higher), household income (<2,500, 2,500-4,999, 5,000-9,999, 10,000-19,999, 20,000-34,999, or ≥35,000 yuan/year), marital status (married, widowed, divorced or separated, or never married), alcohol consumption (not weekly; ex-regular; not daily; daily consuming 1-15, 15-29, 30-59, or ≥60 g), tobacco smoking (never or occasional; former; current smoking with 1-14, 15-24, or ≥25 cigarettes/day), physical activity in MET-hours/day (continuous), BMI (continuous), waist to hip ratio (continuous), prevalent hypertension (presence or absence), use of aspirin (presence, absence, or unknown), family history of CVD (presence or absence); model 3: additionally included intake of multivitamin supplementation (presence or absence) and dietary pattern (new affluence, traditional northern, or traditional southern), as appropriate.

* No. of major coronary events.

† The tests for interaction were performed using likelihood ratio tests, which involved comparing models with and without interaction terms between the strata variable and egg consumption (as a multinomial variable).

**Supplemental Table S9. Subgroup analysis of associations between egg consumption and risk of hemorrhagic stroke according to potential baseline risk factors.**

| **Subgroups** | **Egg consumption** | | | | | | | | | | | | | | |
| --- | --- | --- | --- | --- | --- | --- | --- | --- | --- | --- | --- | --- | --- | --- | --- |
|  | **Never/rarely** | |  | **1-3 days/month** | |  | **1-3 d/week** | |  | **4-6 d/week** | |  | **7 d/week** | | ***P* for interaction†** |
|  | **No.*** | **HR** |  | **No.*** | **HR(%95CI)** |  | **No.*** | **HR(%95CI)** |  | **No.*** | **HR(%95CI)** |  | **No.*** | **HR(%95CI)** |  |
| **Sex** |  |  |  |  |  |  |  |  |  |  |  |  |  |  |  |
| Women | 544 | 1.00 |  | 776 | 0.84 (0.75, 0.94) |  | 1,324 | 0.78 (0.70, 0.87) |  | 339 | 0.77 (0.67, 0.89) |  | 284 | 0.71 (0.61, 0.83) | 0.137 |
| Men | 409 | 1.00 |  | 789 | 0.90 (0.79, 1.01) |  | 1,756 | 0.88 (0.78, 0.98) |  | 418 | 0.79 (0.69, 0.92) |  | 439 | 0.79 (0.68, 0.91) |  |
| **Age (yr)** |  |  |  |  |  |  |  |  |  |  |  |  |  |  |  |
| <50 | 143 | 1.00 |  | 238 | 0.80 (0.65, 0.99) |  | 590 | 0.81 (0.67, 0.98) |  | 161 | 0.81 (0.64, 1.03) |  | 132 | 0.73 (0.57, 0.95) | 0.868 |
| 50-60 | 273 | 1.00 |  | 461 | 0.88 (0.75, 1.02) |  | 909 | 0.83 (0.72, 0.96) |  | 239 | 0.77 (0.64, 0.93) |  | 218 | 0.83 (0.69, 1.01) |  |
| ≥60 | 537 | 1.00 |  | 866 | 0.86 (0.77, 0.96) |  | 1,581 | 0.82 (0.74, 0.91) |  | 357 | 0.76 (0.66, 0.88) |  | 373 | 0.70 (0.61, 0.81) |  |
| **Area** |  |  |  |  |  |  |  |  |  |  |  |  |  |  |  |
| Urban | 145 | 1.00 |  | 259 | 0.87 (0.71, 1.07) |  | 887 | 0.92 (0.77, 1.11) |  | 111 | 0.81 (0.62, 1.05) |  | 289 | 0.90 (0.71, 1.13) | 0.048 |
| Rural | 808 | 1.00 |  | 1,306 | 0.85 (0.78, 0.93) |  | 2,193 | 0.80 (0.73, 0.87) |  | 646 | 0.77 (0.69, 0.86) |  | 434 | 0.71 (0.63, 0.80) |  |
| **Highest education level** |  |  |  |  |  |  |  |  |  |  |  |  |  |  |  |
| Primary school and below | 786 | 1.00 |  | 1,274 | 0.86 (0.79, 0.94) |  | 2,110 | 0.79 (0.72, 0.86) |  | 531 | 0.75 (0.67, 0.84) |  | 388 | 0.69 (0.61, 0.79) | 0.336 |
| Middle or high school | 160 | 1.00 |  | 273 | 0.83 (0.68, 1.01) |  | 883 | 0.89 (0.75, 1.07) |  | 207 | 0.83 (0.67, 1.03) |  | 288 | 0.84 (0.69, 1.04) |  |
| College and above | 7 | 1.00 |  | 18 | 1.11 (0.45, 2.75) |  | 87 | 1.20 (0.53, 2.72) |  | 19 | 1.22 (0.49, 3.06) |  | 47 | 1.25 (0.53, 2.96) |  |
| **Household income (yuan/yr)** |  |  |  |  |  |  |  |  |  |  |  |  |  |  |  |
| <10,000 | 622 | 1.00 |  | 866 | 0.86 (0.77, 0.96) |  | 1,164 | 0.81 (0.73, 0.90) |  | 226 | 0.66 (0.57, 0.77) |  | 261 | 0.67 (0.58, 0.78) | 0.093 |
| 10,000-19,999 | 221 | 1.00 |  | 370 | 0.86 (0.72, 1.02) |  | 952 | 0.84 (0.72, 0.98) |  | 259 | 0.89 (0.73, 1.08) |  | 228 | 0.81 (0.66, 0.99) |  |
| ≥20,000 | 110 | 1.00 |  | 329 | 0.86 (0.69, 1.07) |  | 964 | 0.85 (0.70, 1.05) |  | 272 | 0.86 (0.68, 1.08) |  | 234 | 0.84 (0.66, 1.07) |  |
| **Alcohol consumption** |  |  |  |  |  |  |  |  |  |  |  |  |  |  |  |
| Not current | 830 | 1.00 |  | 1,331 | 0.83 (0.76, 0.91) |  | 2,503 | 0.80 (0.74, 0.87) |  | 646 | 0.76 (0.68, 0.85) |  | 549 | 0.73 (0.65, 0.82) | 0.624 |
| Current | 123 | 1.00 |  | 234 | 1.00 (0.80, 1.24) |  | 577 | 0.92 (0.75, 1.14) |  | 111 | 0.84 (0.64, 1.10) |  | 174 | 0.86 (0.67, 1.10) |  |
| **Tobacco smoking** |  |  |  |  |  |  |  |  |  |  |  |  |  |  |  |
| Not current | 632 | 1.00 |  | 959 | 0.80 (0.73, 0.89) |  | 1,790 | 0.75 (0.68, 0.83) |  | 459 | 0.75 (0.66, 0.85) |  | 413 | 0.71 (0.62, 0.82) | 0.004 |
| Current | 321 | 1.00 |  | 606 | 0.97 (0.84, 1.11) |  | 1,290 | 0.96 (0.84, 1.09) |  | 298 | 0.83 (0.70, 0.98) |  | 310 | 0.81 (0.69, 0.96) |  |
| **Physical activity (MET-hr/day)** |  |  |  |  |  |  |  |  |  |  |  |  |  |  |  |
| Low | 461 | 1.00 |  | 692 | 0.84 (0.75, 0.95) |  | 1,547 | 0.81 (0.73, 0.91) |  | 340 | 0.76 (0.65, 0.88) |  | 394 | 0.76 (0.65, 0.88) | 0.578 |
| Middle | 300 | 1.00 |  | 496 | 0.86 (0.74, 1.00) |  | 848 | 0.79 (0.68, 0.91) |  | 217 | 0.68 (0.57, 0.83) |  | 192 | 0.69 (0.56, 0.83) |  |
| High | 192 | 1.00 |  | 377 | 0.89 (0.75, 1.07) |  | 685 | 0.87 (0.74, 1.03) |  | 200 | 0.93 (0.75, 1.14) |  | 137 | 0.77 (0.61, 0.97) |  |
| **Dietary pattern** |  |  |  |  |  |  |  |  |  |  |  |  |  |  |  |
| New affluence | 20 | 1.00 |  | 39 | 1.01 (0.59, 1.74) |  | 343 | 1.23 (0.78, 1.94) |  | 144 | 1.18 (0.73, 1.90) |  | 328 | 1.11 (0.71, 1.76) | 0.303 |
| Traditional southern | 507 | 1.00 |  | 843 | 0.84 (0.75, 0.95) |  | 1,886 | 0.81 (0.72, 0.90) |  | 382 | 0.75 (0.65, 0.87) |  | 148 | 0.77 (0.64, 0.93) |  |
| Traditional northern | 426 | 1.00 |  | 683 | 0.86 (0.76, 0.97) |  | 851 | 0.81 (0.72, 0.92) |  | 231 | 0.78 (0.67, 0.92) |  | 247 | 0.73 (0.62, 0.85) |  |
| **BMI (kg/m^2^)** |  |  |  |  |  |  |  |  |  |  |  |  |  |  |  |
| <24.0 | 544 | 1.00 |  | 1,024 | 0.93 (0.83, 1.03) |  | 1,862 | 0.86 (0.77, 0.95) |  | 506 | 0.83 (0.73, 0.95) |  | 404 | 0.79 (0.69, 0.91) | 0.444 |
| 24.0-28.0 | 295 | 1.00 |  | 405 | 0.75 (0.64, 0.88) |  | 901 | 0.74 (0.64, 0.85) |  | 185 | 0.64 (0.53, 0.78) |  | 231 | 0.67 (0.55, 0.81) |  |
| ≥28.0 | 114 | 1.00 |  | 136 | 0.79 (0.61, 1.02) |  | 317 | 0.87 (0.69, 1.10) |  | 66 | 0.87 (0.63, 1.20) |  | 88 | 0.74 (0.54, 1.02) |  |
| **Hypertension** |  |  |  |  |  |  |  |  |  |  |  |  |  |  |  |
| Yes | 221 | 1.00 |  | 399 | 0.86 (0.73, 1.02) |  | 875 | 0.85 (0.72, 0.99) |  | 227 | 0.77 (0.63, 0.94) |  | 244 | 0.84 (0.69, 1.02) | 0.531 |
| No | 732 | 1.00 |  | 1,166 | 0.86 (0.78, 0.94) |  | 2,205 | 0.82 (0.75, 0.89) |  | 530 | 0.78 (0.70, 0.88) |  | 479 | 0.71 (0.63, 0.81) |  |

HR indicates hazard ratios; CI confidence interval.

Stratified Cox proportional models were used with stratification on survey site and birth cohort (in 5-year intervals), except for subgroup and interaction analyses according to age at recruitment (only with stratification on survey site). Multivariate models were adjusted for age at recruitment (continuous) and sex (men or women); model 2: additionally included education level (no formal school, primary school, middle school, high school, college, or university or higher), household income (<2,500, 2,500-4,999, 5,000-9,999, 10,000-19,999, 20,000-34,999, or ≥35,000 yuan/year), marital status (married, widowed, divorced or separated, or never married), alcohol consumption (not weekly; ex-regular; not daily; daily consuming 1-15, 15-29, 30-59, or ≥60 g), tobacco smoking (never or occasional; former; current smoking with 1-14, 15-24, or ≥25 cigarettes/day), physical activity in MET-hours/day (continuous), BMI (continuous), waist to hip ratio (continuous), prevalent hypertension (presence or absence), use of aspirin (presence, absence, or unknown), family history of CVD (presence or absence); model 3: additionally included intake of multivitamin supplementation (presence or absence) and dietary pattern (new affluence, traditional northern, or traditional southern), as appropriate.

* No. of incident hemorrhagic stroke cases.

† The tests for interaction were performed using likelihood ratio tests, which involved comparing models with and without interaction terms between the strata variable and egg consumption (as a multinomial variable).

**Supplemental Table S10. Subgroup analysis of associations between egg consumption and risk of ischemic stroke according to potential baseline risk factors.**

| **Subgroups** | **Egg consumption** | | | | | | | | | | | | | | |
| --- | --- | --- | --- | --- | --- | --- | --- | --- | --- | --- | --- | --- | --- | --- | --- |
|  | **Never/rarely** | |  | **1-3 days/month** | |  | **1-3 d/week** | |  | **4-6 d/week** | |  | **7 d/week** | | ***P* for interaction†** |
|  | **No.*** | **HR** |  | **No.*** | **HR(%95CI)** |  | **No.*** | **HR(%95CI)** |  | **No.*** | **HR(%95CI)** |  | **No.*** | **HR(%95CI)** |  |
| **Sex** |  |  |  |  |  |  |  |  |  |  |  |  |  |  |  |
| Women | 1,826 | 1.00 |  | 3,167 | 0.97 (0.92, 1.03) |  | 6,490 | 0.94 (0.89, 0.99) |  | 1,324 | 0.93 (0.86, 1.00) |  | 2,123 | 0.90 (0.84, 0.96) | 0.137 |
| Men | 1,014 | 1.00 |  | 2,347 | 1.00 (0.93, 1.08) |  | 6,130 | 0.99 (0.92, 1.06) |  | 1,289 | 0.99 (0.91, 1.08) |  | 2,035 | 0.91 (0.84, 0.98) |  |
| **Age (yr)** |  |  |  |  |  |  |  |  |  |  |  |  |  |  |  |
| <50 | 421 | 1.00 |  | 901 | 0.97 (0.87, 1.09) |  | 2,442 | 0.97 (0.87, 1.08) |  | 603 | 1.04 (0.91, 1.18) |  | 734 | 0.94 (0.83, 1.07) | 0.292 |
| 50-60 | 982 | 1.00 |  | 1,851 | 0.97 (0.90, 1.05) |  | 4,158 | 0.93 (0.87, 1.00) |  | 880 | 0.92 (0.84, 1.02) |  | 1,236 | 0.88 (0.80, 0.96) |  |
| ≥60 | 1,437 | 1.00 |  | 2,762 | 0.98 (0.92, 1.05) |  | 6,020 | 0.96 (0.91, 1.02) |  | 1,130 | 0.93 (0.85, 1.01) |  | 2,188 | 0.87 (0.81, 0.94) |  |
| **Area** |  |  |  |  |  |  |  |  |  |  |  |  |  |  |  |
| Urban | 1,219 | 1.00 |  | 2,324 | 0.95 (0.89, 1.02) |  | 6,618 | 0.94 (0.88, 1.00) |  | 1,138 | 0.95 (0.87, 1.04) |  | 2,716 | 0.88 (0.81, 0.95) | 0.739 |
| Rural | 1,621 | 1.00 |  | 3,190 | 1.01 (0.95, 1.07) |  | 6,002 | 0.98 (0.93, 1.04) |  | 1,475 | 0.96 (0.89, 1.03) |  | 1,442 | 0.93 (0.87, 1.00) |  |
| **Highest education level** |  |  |  |  |  |  |  |  |  |  |  |  |  |  |  |
| Primary school and below | 1,959 | 1.00 |  | 3,611 | 0.97 (0.92, 1.02) |  | 6,777 | 0.96 (0.91, 1.01) |  | 1,356 | 0.93 (0.86, 1.00) |  | 1,616 | 0.90 (0.84, 0.97) | 0.075 |
| Middle or high school | 808 | 1.00 |  | 1,710 | 1.00 (0.92, 1.08) |  | 4,945 | 0.93 (0.86, 1.01) |  | 1,073 | 0.99 (0.90, 1.08) |  | 2,031 | 0.91 (0.84, 1.00) |  |
| College and above | 73 | 1.00 |  | 193 | 1.02 (0.78, 1.34) |  | 898 | 1.01 (0.79, 1.29) |  | 184 | 0.89 (0.67, 1.18) |  | 511 | 0.81 (0.62, 1.04) |  |
| **Household income (yuan/yr)** |  |  |  |  |  |  |  |  |  |  |  |  |  |  |  |
| <10,000 | 1,374 | 1.00 |  | 2,181 | 0.97 (0.91, 1.04) |  | 3,515 | 0.95 (0.89, 1.02) |  | 602 | 0.89 (0.81, 0.99) |  | 984 | 0.90 (0.82, 0.98) | 0.012 |
| 10,000-19,999 | 886 | 1.00 |  | 1,582 | 0.94 (0.86, 1.02) |  | 4,252 | 0.96 (0.89, 1.04) |  | 857 | 0.98 (0.89, 1.08) |  | 1,429 | 0.94 (0.86, 1.03) |  |
| ≥20,000 | 580 | 1.00 |  | 1,751 | 1.05 (0.95, 1.15) |  | 4,853 | 0.97 (0.89, 1.06) |  | 1,154 | 0.96 (0.87, 1.07) |  | 1,745 | 0.87 (0.79, 0.97) |  |
| **Alcohol consumption** |  |  |  |  |  |  |  |  |  |  |  |  |  |  |  |
| Not current | 2,539 | 1.00 |  | 4,857 | 0.98 (0.93, 1.03) |  | 10,504 | 0.96 (0.91, 1.00) |  | 2,174 | 0.94 (0.88, 1.00) |  | 3,316 | 0.90 (0.85, 0.96) | 0.731 |
| Current | 301 | 1.00 |  | 657 | 0.95 (0.83, 1.09) |  | 2,116 | 0.93 (0.82, 1.05) |  | 439 | 0.99 (0.85, 1.15) |  | 842 | 0.87 (0.76, 1.00) |  |
| **Tobacco smoking** |  |  |  |  |  |  |  |  |  |  |  |  |  |  |  |
| Not current | 2,059 | 1.00 |  | 3,855 | 0.98 (0.93, 1.03) |  | 8,322 | 0.94 (0.90, 0.99) |  | 1,695 | 0.94 (0.88, 1.00) |  | 2,750 | 0.90 (0.84, 0.96) | 0.058 |
| Current | 781 | 1.00 |  | 1,659 | 0.99 (0.91, 1.08) |  | 4,298 | 0.98 (0.91, 1.06) |  | 918 | 0.98 (0.89, 1.09) |  | 1,408 | 0.90 (0.82, 0.99) |  |
| **Physical activity (MET-hr/day)** |  |  |  |  |  |  |  |  |  |  |  |  |  |  |  |
| Low | 1,563 | 1.00 |  | 2,943 | 1.01 (0.95, 1.07) |  | 6,935 | 0.96 (0.91, 1.02) |  | 1,322 | 0.97 (0.89, 1.04) |  | 2,448 | 0.89 (0.83, 0.95) | 0.753 |
| Middle | 812 | 1.00 |  | 1,541 | 0.96 (0.88, 1.04) |  | 3,414 | 0.95 (0.88, 1.03) |  | 755 | 0.92 (0.83, 1.02) |  | 1,111 | 0.91 (0.83, 1.01) |  |
| High | 465 | 1.00 |  | 1,030 | 0.94 (0.84, 1.05) |  | 2,271 | 0.94 (0.84, 1.04) |  | 536 | 0.96 (0.84, 1.09) |  | 599 | 0.94 (0.82, 1.07) |  |
| **Dietary pattern** |  |  |  |  |  |  |  |  |  |  |  |  |  |  |  |
| New affluence | 210 | 1.00 |  | 456 | 1.05 (0.89, 1.24) |  | 2,877 | 0.97 (0.84, 1.12) |  | 1,018 | 1.01 (0.87, 1.17) |  | 2,637 | 0.87 (0.76, 1.01) | 0.038 |
| Traditional southern | 1,560 | 1.00 |  | 3,125 | 0.93 (0.87, 0.99) |  | 6,302 | 0.90 (0.85, 0.96) |  | 933 | 0.87 (0.80, 0.95) |  | 369 | 0.91 (0.81, 1.02) |  |
| Traditional northern | 1,070 | 1.00 |  | 1,933 | 1.03 (0.95, 1.11) |  | 3,441 | 1.01 (0.94, 1.08) |  | 662 | 0.98 (0.89, 1.08) |  | 1,152 | 1.00 (0.91, 1.08) |  |
| **BMI (kg/m^2^)** |  |  |  |  |  |  |  |  |  |  |  |  |  |  |  |
| <24.0 | 1,420 | 1.00 |  | 2,916 | 1.00 (0.93, 1.06) |  | 6,187 | 0.96 (0.90, 1.02) |  | 1,364 | 0.96 (0.88, 1.04) |  | 1,838 | 0.89 (0.82, 0.96) | 0.852 |
| 24.0-28.0 | 988 | 1.00 |  | 1,944 | 0.99 (0.92, 1.07) |  | 4,681 | 0.95 (0.89, 1.02) |  | 934 | 0.96 (0.87, 1.06) |  | 1,646 | 0.91 (0.84, 1.00) |  |
| ≥28.0 | 432 | 1.00 |  | 654 | 0.91 (0.80, 1.02) |  | 1,752 | 0.94 (0.84, 1.05) |  | 315 | 0.91 (0.78, 1.06) |  | 674 | 0.89 (0.78, 1.02) |  |
| **Hypertension** |  |  |  |  |  |  |  |  |  |  |  |  |  |  |  |
| Yes | 1,105 | 1.00 |  | 2,256 | 1.00 (0.93, 1.07) |  | 5,226 | 0.96 (0.90, 1.03) |  | 1,160 | 0.95 (0.87, 1.03) |  | 1,999 | 0.94 (0.87, 1.02) | <0.001 |
| No | 1,735 | 1.00 |  | 3,258 | 0.98 (0.92, 1.04) |  | 7,394 | 0.96 (0.91, 1.02) |  | 1,453 | 0.97 (0.90, 1.04) |  | 2,159 | 0.88 (0.82, 0.94) |  |

HR indicates hazard ratios; CI confidence interval.

Stratified Cox proportional models were used with stratification on survey site and birth cohort (in 5-year intervals), except for subgroup and interaction analyses according to age at recruitment (only with stratification on survey site). Multivariate models were adjusted for age at recruitment (continuous) and sex (men or women); model 2: additionally included education level (no formal school, primary school, middle school, high school, college, or university or higher), household income (<2,500, 2,500-4,999, 5,000-9,999, 10,000-19,999, 20,000-34,999, or ≥35,000 yuan/year), marital status (married, widowed, divorced or separated, or never married), alcohol consumption (not weekly; ex-regular; not daily; daily consuming 1-15, 15-29, 30-59, or ≥60 g), tobacco smoking (never or occasional; former; current smoking with 1-14, 15-24, or ≥25 cigarettes/day), physical activity in MET-hours/day (continuous), BMI (continuous), waist to hip ratio (continuous), prevalent hypertension (presence or absence), use of aspirin (presence, absence, or unknown), family history of CVD (presence or absence); model 3: additionally included intake of multivitamin supplementation (presence or absence) and dietary pattern (new affluence, traditional northern, or traditional southern), as appropriate.

* No. of incident ischemic stroke cases.

† The tests for interaction were performed using likelihood ratio tests, which involved comparing models with and without interaction terms between the strata variable and egg consumption (as a multinomial variable).

**Supplemental Table S11. Subgroup analysis of associations between egg consumption and mortality from cardiovascular disease according to potential baseline risk factors.**

| **Subgroups** | **Egg consumption** | | | | | | | | | | | | | | |
| --- | --- | --- | --- | --- | --- | --- | --- | --- | --- | --- | --- | --- | --- | --- | --- |
|  | **Never/rarely** | |  | **1-3 days/month** | |  | **1-3 d/week** | |  | **4-6 d/week** | |  | **7 d/week** | | ***P* for interaction†** |
|  | **No.*** | **HR** |  | **No.*** | **HR(%95CI)** |  | **No.*** | **HR(%95CI)** |  | **No.*** | **HR(%95CI)** |  | **No.*** | **HR(%95CI)** |  |
| **Sex** |  |  |  |  |  |  |  |  |  |  |  |  |  |  |  |
| Women | 709 | 1.00 |  | 1,048 | 0.91 (0.83, 1.01) |  | 1,736 | 0.88 (0.80, 0.97) |  | 362 | 0.80 (0.70, 0.91) |  | 450 | 0.85 (0.75, 0.97) | 0.434 |
| Men | 607 | 1.00 |  | 1,186 | 0.91 (0.83, 1.01) |  | 2,560 | 0.88 (0.81, 0.97) |  | 573 | 0.80 (0.71, 0.90) |  | 754 | 0.81 (0.73, 0.91) |  |
| **Age (yr)** |  |  |  |  |  |  |  |  |  |  |  |  |  |  |  |
| <50 | 117 | 1.00 |  | 216 | 0.94 (0.74, 1.18) |  | 430 | 0.89 (0.72, 1.10) |  | 114 | 0.95 (0.73, 1.25) |  | 126 | 0.96 (0.73, 1.27) | 0.410 |
| 50-60 | 265 | 1.00 |  | 463 | 0.96 (0.83, 1.12) |  | 828 | 0.86 (0.74, 1.00) |  | 187 | 0.71 (0.59, 0.87) |  | 228 | 0.88 (0.72, 1.06) |  |
| ≥60 | 934 | 1.00 |  | 1,555 | 0.89 (0.82, 0.97) |  | 3,038 | 0.89 (0.82, 0.96) |  | 634 | 0.80 (0.72, 0.89) |  | 850 | 0.78 (0.70, 0.86) |  |
| **Area** |  |  |  |  |  |  |  |  |  |  |  |  |  |  |  |
| Urban | 236 | 1.00 |  | 422 | 0.97 (0.82, 1.13) |  | 1,361 | 1.02 (0.88, 1.18) |  | 183 | 0.94 (0.77, 1.15) |  | 539 | 1.05 (0.88, 1.26) | 0.001 |
| Rural | 1,080 | 1.00 |  | 1,812 | 0.90 (0.83, 0.97) |  | 2,935 | 0.85 (0.79, 0.92) |  | 752 | 0.77 (0.70, 0.86) |  | 665 | 0.77 (0.70, 0.85) |  |
| **Highest education level** |  |  |  |  |  |  |  |  |  |  |  |  |  |  |  |
| Primary school and below | 1,113 | 1.00 |  | 1,855 | 0.90 (0.84, 0.97) |  | 3,211 | 0.87 (0.81, 0.94) |  | 698 | 0.78 (0.71, 0.86) |  | 721 | 0.78 (0.71, 0.86) | 0.526 |
| Middle or high school | 197 | 1.00 |  | 361 | 0.94 (0.79, 1.12) |  | 975 | 0.87 (0.74, 1.02) |  | 213 | 0.80 (0.65, 0.98) |  | 409 | 0.88 (0.73, 1.06) |  |
| College and above | 6 | 1.00 |  | 18 | 0.90 (0.35, 2.30) |  | 110 | 1.32 (0.56, 3.08) |  | 24 | 1.17 (0.46, 3.01) |  | 74 | 1.39 (0.57, 3.35) |  |
| **Household income (yuan/yr)** |  |  |  |  |  |  |  |  |  |  |  |  |  |  |  |
| <10,000 | 914 | 1.00 |  | 1,331 | 0.88 (0.81, 0.96) |  | 1,928 | 0.86 (0.79, 0.93) |  | 370 | 0.74 (0.65, 0.84) |  | 477 | 0.71 (0.63, 0.80) | 0.038 |
| 10,000-19,999 | 278 | 1.00 |  | 509 | 0.97 (0.84, 1.13) |  | 1,290 | 0.95 (0.83, 1.09) |  | 287 | 0.88 (0.74, 1.05) |  | 389 | 1.01 (0.85, 1.19) |  |
| ≥20,000 | 124 | 1.00 |  | 394 | 0.98 (0.80, 1.20) |  | 1,078 | 0.93 (0.77, 1.13) |  | 278 | 0.90 (0.72, 1.12) |  | 338 | 0.94 (0.75, 1.18) |  |
| **Alcohol consumption** |  |  |  |  |  |  |  |  |  |  |  |  |  |  |  |
| Not current | 1,143 | 1.00 |  | 1,937 | 0.91 (0.85, 0.98) |  | 3,591 | 0.89 (0.83, 0.96) |  | 790 | 0.79 (0.72, 0.87) |  | 946 | 0.82 (0.74, 0.90) | 0.843 |
| Current | 173 | 1.00 |  | 297 | 0.89 (0.73, 1.08) |  | 705 | 0.80 (0.67, 0.96) |  | 145 | 0.82 (0.65, 1.03) |  | 258 | 0.82 (0.66, 1.01) |  |
| **Tobacco smoking** |  |  |  |  |  |  |  |  |  |  |  |  |  |  |  |
| Not current | 810 | 1.00 |  | 1,311 | 0.89 (0.82, 0.98) |  | 2,411 | 0.86 (0.79, 0.94) |  | 498 | 0.77 (0.69, 0.87) |  | 635 | 0.82 (0.73, 0.92) | 0.223 |
| Current | 506 | 1.00 |  | 923 | 0.95 (0.85, 1.06) |  | 1,885 | 0.92 (0.83, 1.02) |  | 437 | 0.84 (0.73, 0.96) |  | 569 | 0.84 (0.73, 0.95) |  |
| **Physical activity (MET-hr/day)** |  |  |  |  |  |  |  |  |  |  |  |  |  |  |  |
| Low | 760 | 1.00 |  | 1,208 | 0.89 (0.81, 0.98) |  | 2,683 | 0.88 (0.81, 0.96) |  | 514 | 0.77 (0.69, 0.87) |  | 769 | 0.78 (0.70, 0.87) | 0.944 |
| Middle | 354 | 1.00 |  | 617 | 0.91 (0.79, 1.04) |  | 977 | 0.86 (0.76, 0.98) |  | 251 | 0.80 (0.67, 0.95) |  | 277 | 0.87 (0.73, 1.03) |  |
| High | 202 | 1.00 |  | 409 | 0.99 (0.83, 1.18) |  | 636 | 0.89 (0.76, 1.06) |  | 170 | 0.86 (0.70, 1.07) |  | 158 | 0.90 (0.72, 1.13) |  |
| **Dietary pattern** |  |  |  |  |  |  |  |  |  |  |  |  |  |  |  |
| New affluence | 37 | 1.00 |  | 73 | 1.08 (0.72, 1.61) |  | 473 | 0.98 (0.70, 1.38) |  | 178 | 0.96 (0.67, 1.38) |  | 585 | 1.06 (0.75, 1.48) | 0.073 |
| Traditional southern | 688 | 1.00 |  | 1,128 | 0.89 (0.80, 0.98) |  | 2,530 | 0.88 (0.81, 0.97) |  | 432 | 0.78 (0.68, 0.88) |  | 203 | 0.83 (0.71, 0.97) |  |
| Traditional northern | 591 | 1.00 |  | 1,033 | 0.92 (0.83, 1.02) |  | 1,293 | 0.87 (0.79, 0.96) |  | 325 | 0.84 (0.73, 0.96) |  | 416 | 0.76 (0.67, 0.87) |  |
| **BMI (kg/m^2^)** |  |  |  |  |  |  |  |  |  |  |  |  |  |  |  |
| <24.0 | 827 | 1.00 |  | 1,518 | 0.94 (0.86, 1.03) |  | 2,725 | 0.88 (0.81, 0.96) |  | 637 | 0.81 (0.72, 0.90) |  | 677 | 0.82 (0.73, 0.91) | 0.169 |
| 24.0-28.0 | 344 | 1.00 |  | 528 | 0.86 (0.75, 0.99) |  | 1,163 | 0.88 (0.77, 1.00) |  | 220 | 0.76 (0.64, 0.91) |  | 394 | 0.88 (0.75, 1.03) |  |
| ≥28.0 | 145 | 1.00 |  | 188 | 0.84 (0.67, 1.05) |  | 408 | 0.83 (0.68, 1.02) |  | 78 | 0.78 (0.58, 1.04) |  | 133 | 0.68 (0.52, 0.89) |  |
| **Hypertension** |  |  |  |  |  |  |  |  |  |  |  |  |  |  |  |
| Yes | 356 | 1.00 |  | 663 | 0.96 (0.85, 1.10) |  | 1,330 | 0.93 (0.82, 1.05) |  | 322 | 0.87 (0.75, 1.03) |  | 429 | 0.88 (0.75, 1.03) | 0.822 |
| No | 960 | 1.00 |  | 1,571 | 0.89 (0.82, 0.97) |  | 2,966 | 0.87 (0.80, 0.94) |  | 613 | 0.77 (0.69, 0.86) |  | 775 | 0.80 (0.72, 0.89) |  |

HR indicates hazard ratios; CI confidence interval.

Stratified Cox proportional models were used with stratification on survey site and birth cohort (in 5-year intervals), except for subgroup and interaction analyses according to age at recruitment (only with stratification on survey site). Multivariate models were adjusted for age at recruitment (continuous) and sex (men or women); model 2: additionally included education level (no formal school, primary school, middle school, high school, college, or university or higher), household income (<2,500, 2,500-4,999, 5,000-9,999, 10,000-19,999, 20,000-34,999, or ≥35,000 yuan/year), marital status (married, widowed, divorced or separated, or never married), alcohol consumption (not weekly; ex-regular; not daily; daily consuming 1-15, 15-29, 30-59, or ≥60 g), tobacco smoking (never or occasional; former; current smoking with 1-14, 15-24, or ≥25 cigarettes/day), physical activity in MET-hours/day (continuous), BMI (continuous), waist to hip ratio (continuous), prevalent hypertension (presence or absence), use of aspirin (presence, absence, or unknown), family history of CVD (presence or absence); model 3: additionally included intake of multivitamin supplementation (presence or absence) and dietary pattern (new affluence, traditional northern, or traditional southern), as appropriate.

* No. of cardiovascular disease deaths.

† The tests for interaction were performed using likelihood ratio tests, which involved comparing models with and without interaction terms between the strata variable and egg consumption (as a multinomial variable).

**Supplemental Table S12. Subgroup analysis of associations between egg consumption and mortality from ischemic heart disease according to potential baseline risk factors.**

| **Subgroups** | **Egg consumption** | | | | | | | | | | | | | | |
| --- | --- | --- | --- | --- | --- | --- | --- | --- | --- | --- | --- | --- | --- | --- | --- |
|  | **Never/rarely** | |  | **1-3 days/month** | |  | **1-3 d/week** | |  | **4-6 d/week** | |  | **7 d/week** | | ***P* for interaction†** |
|  | **No.*** | **HR** |  | **No.*** | **HR(%95CI)** |  | **No.*** | **HR(%95CI)** |  | **No.*** | **HR(%95CI)** |  | **No.*** | **HR(%95CI)** |  |
| **Sex** |  |  |  |  |  |  |  |  |  |  |  |  |  |  |  |
| Women | 210 | 1.00 |  | 326 | 0.95 (0.80, 1.14) |  | 577 | 0.93 (0.79, 1.10) |  | 136 | 0.92 (0.73, 1.16) |  | 188 | 0.93 (0.74, 1.15) | 0.214 |
| Men | 185 | 1.00 |  | 338 | 0.85 (0.71, 1.02) |  | 885 | 0.94 (0.79, 1.11) |  | 202 | 0.81 (0.66, 1.00) |  | 327 | 0.87 (0.72, 1.06) |  |
| **Age (yr)** |  |  |  |  |  |  |  |  |  |  |  |  |  |  |  |
| <50 | 27 | 1.00 |  | 74 | 1.33 (0.85, 2.08) |  | 154 | 1.28 (0.83, 1.96) |  | 39 | 1.21 (0.72, 2.02) |  | 54 | 1.41 (0.86, 2.32) | 0.825 |
| 50-60 | 80 | 1.00 |  | 120 | 0.81 (0.61, 1.08) |  | 269 | 0.87 (0.67, 1.13) |  | 63 | 0.76 (0.54, 1.08) |  | 81 | 0.81 (0.58, 1.14) |  |
| ≥60 | 288 | 1.00 |  | 470 | 0.89 (0.76, 1.03) |  | 1,039 | 0.93 (0.81, 1.07) |  | 236 | 0.85 (0.71, 1.02) |  | 380 | 0.86 (0.72, 1.01) |  |
| **Area** |  |  |  |  |  |  |  |  |  |  |  |  |  |  |  |
| Urban | 82 | 1.00 |  | 142 | 0.96 (0.73, 1.27) |  | 530 | 1.15 (0.90, 1.48) |  | 82 | 1.03 (0.74, 1.43) |  | 267 | 1.06 (0.80, 1.40) | 0.199 |
| Rural | 313 | 1.00 |  | 522 | 0.88 (0.76, 1.01) |  | 932 | 0.87 (0.76, 1.00) |  | 256 | 0.81 (0.68, 0.96) |  | 248 | 0.86 (0.72, 1.02) |  |
| **Highest education level** |  |  |  |  |  |  |  |  |  |  |  |  |  |  |  |
| Primary school and below | 321 | 1.00 |  | 530 | 0.90 (0.78, 1.03) |  | 1,036 | 0.95 (0.83, 1.08) |  | 237 | 0.84 (0.71, 1.01) |  | 296 | 0.91 (0.76, 1.07) | 0.961 |
| Middle or high school | 71 | 1.00 |  | 126 | 0.90 (0.67, 1.21) |  | 370 | 0.86 (0.66, 1.13) |  | 91 | 0.85 (0.61, 1.18) |  | 177 | 0.81 (0.60, 1.09) |  |
| College and above | 3 | 1.00 |  | 8 | 0.73 (0.19, 2.82) |  | 56 | 1.09 (0.33, 3.58) |  | 10 | 0.64 (0.16, 2.51) |  | 42 | 1.09 (0.32, 3.71) |  |
| **Household income (yuan/yr)** |  |  |  |  |  |  |  |  |  |  |  |  |  |  |  |
| <10,000 | 278 | 1.00 |  | 403 | 0.86 (0.73, 1.00) |  | 609 | 0.84 (0.72, 0.97) |  | 139 | 0.84 (0.68, 1.03) |  | 187 | 0.78 (0.64, 0.95) | 0.336 |
| 10,000-19,999 | 80 | 1.00 |  | 154 | 1.03 (0.79, 1.36) |  | 461 | 1.10 (0.86, 1.41) |  | 96 | 0.96 (0.70, 1.31) |  | 177 | 1.24 (0.93, 1.66) |  |
| ≥20,000 | 37 | 1.00 |  | 107 | 1.06 (0.72, 1.56) |  | 392 | 1.20 (0.84, 1.70) |  | 103 | 1.01 (0.68, 1.50) |  | 151 | 0.99 (0.67, 1.46) |  |
| **Alcohol consumption** |  |  |  |  |  |  |  |  |  |  |  |  |  |  |  |
| Not current | 343 | 1.00 |  | 596 | 0.94 (0.82, 1.07) |  | 1,243 | 0.99 (0.87, 1.12) |  | 286 | 0.89 (0.75, 1.05) |  | 404 | 0.93 (0.79, 1.09) | 0.372 |
| Current | 52 | 1.00 |  | 68 | 0.66 (0.46, 0.96) |  | 219 | 0.66 (0.48, 0.91) |  | 52 | 0.66 (0.44, 1.00) |  | 111 | 0.68 (0.47, 0.98) |  |
| **Tobacco smoking** |  |  |  |  |  |  |  |  |  |  |  |  |  |  |  |
| Not current | 238 | 1.00 |  | 400 | 0.93 (0.79, 1.09) |  | 824 | 0.95 (0.82, 1.11) |  | 179 | 0.86 (0.70, 1.06) |  | 273 | 0.92 (0.76, 1.11) | 0.612 |
| Current | 157 | 1.00 |  | 264 | 0.87 (0.71, 1.06) |  | 638 | 0.92 (0.76, 1.10) |  | 159 | 0.85 (0.67, 1.07) |  | 242 | 0.86 (0.69, 1.07) |  |
| **Physical activity (MET-hr/day)** |  |  |  |  |  |  |  |  |  |  |  |  |  |  |  |
| Low | 247 | 1.00 |  | 385 | 0.87 (0.74, 1.02) |  | 978 | 0.93 (0.80, 1.07) |  | 185 | 0.77 (0.63, 0.94) |  | 342 | 0.81 (0.67, 0.97) | 0.590 |
| Middle | 95 | 1.00 |  | 168 | 0.91 (0.70, 1.17) |  | 299 | 0.93 (0.73, 1.19) |  | 98 | 1.05 (0.78, 1.42) |  | 108 | 1.02 (0.76, 1.39) |  |
| High | 53 | 1.00 |  | 111 | 1.03 (0.74, 1.44) |  | 185 | 0.96 (0.70, 1.33) |  | 55 | 0.90 (0.61, 1.34) |  | 65 | 1.08 (0.73, 1.60) |  |
| **Dietary pattern** |  |  |  |  |  |  |  |  |  |  |  |  |  |  |  |
| New affluence | 20 | 1.00 |  | 33 | 0.89 (0.51, 1.57) |  | 221 | 0.87 (0.54, 1.38) |  | 69 | 0.71 (0.43, 1.18) |  | 287 | 0.87 (0.55, 1.38) | 0.726 |
| Traditional southern | 191 | 1.00 |  | 299 | 0.85 (0.71, 1.03) |  | 770 | 0.92 (0.77, 1.10) |  | 149 | 0.84 (0.66, 1.06) |  | 55 | 0.75 (0.55, 1.02) |  |
| Traditional northern | 184 | 1.00 |  | 332 | 0.94 (0.79, 1.13) |  | 471 | 0.95 (0.80, 1.13) |  | 120 | 0.98 (0.78, 1.24) |  | 173 | 0.95 (0.77, 1.18) |  |
| **BMI (kg/m^2^)** |  |  |  |  |  |  |  |  |  |  |  |  |  |  |  |
| <24.0 | 249 | 1.00 |  | 430 | 0.89 (0.76, 1.04) |  | 896 | 0.93 (0.80, 1.08) |  | 218 | 0.81 (0.67, 0.98) |  | 264 | 0.84 (0.69, 1.01) | 0.859 |
| 24.0-28.0 | 104 | 1.00 |  | 169 | 0.89 (0.70, 1.14) |  | 407 | 0.91 (0.73, 1.14) |  | 94 | 0.98 (0.73, 1.31) |  | 177 | 0.99 (0.76, 1.30) |  |
| ≥28.0 | 42 | 1.00 |  | 65 | 0.99 (0.67, 1.47) |  | 159 | 1.01 (0.71, 1.46) |  | 26 | 0.75 (0.45, 1.26) |  | 74 | 0.91 (0.59, 1.41) |  |
| **Hypertension** |  |  |  |  |  |  |  |  |  |  |  |  |  |  |  |
| Yes | 135 | 1.00 |  | 241 | 0.91 (0.73, 1.12) |  | 504 | 0.86 (0.71, 1.06) |  | 140 | 0.87 (0.68, 1.12) |  | 196 | 0.88 (0.69, 1.12) | 0.334 |
| No | 260 | 1.00 |  | 423 | 0.89 (0.76, 1.05) |  | 958 | 0.98 (0.85, 1.13) |  | 198 | 0.84 (0.69, 1.02) |  | 319 | 0.89 (0.75, 1.07) |  |

HR indicates hazard ratios; CI confidence interval.

Stratified Cox proportional models were used with stratification on survey site and birth cohort (in 5-year intervals), except for subgroup and interaction analyses according to age at recruitment (only with stratification on survey site). Multivariate models were adjusted for age at recruitment (continuous) and sex (men or women); model 2: additionally included education level (no formal school, primary school, middle school, high school, college, or university or higher), household income (<2,500, 2,500-4,999, 5,000-9,999, 10,000-19,999, 20,000-34,999, or ≥35,000 yuan/year), marital status (married, widowed, divorced or separated, or never married), alcohol consumption (not weekly; ex-regular; not daily; daily consuming 1-15, 15-29, 30-59, or ≥60 g), tobacco smoking (never or occasional; former; current smoking with 1-14, 15-24, or ≥25 cigarettes/day), physical activity in MET-hours/day (continuous), BMI (continuous), waist to hip ratio (continuous), prevalent hypertension (presence or absence), use of aspirin (presence, absence, or unknown), family history of CVD (presence or absence); model 3: additionally included intake of multivitamin supplementation (presence or absence) and dietary pattern (new affluence, traditional northern, or traditional southern), as appropriate.

* No. of ischemic heart disease deaths.

† The tests for interaction were performed using likelihood ratio tests, which involved comparing models with and without interaction terms between the strata variable and egg consumption (as a multinomial variable).

**Supplemental Table S13. Subgroup analysis of associations between egg consumption and mortality from hemorrhagic stroke according to potential baseline risk factors.**

| **Subgroups** | **Egg consumption** | | | | | | | | | | | | | | |
| --- | --- | --- | --- | --- | --- | --- | --- | --- | --- | --- | --- | --- | --- | --- | --- |
|  | **Never/rarely** | |  | **1-3 days/month** | |  | **1-3 d/week** | |  | **4-6 d/week** | |  | **7 d/week** | | ***P* for interaction†** |
|  | **No.*** | **HR** |  | **No.*** | **HR(%95CI)** |  | **No.*** | **HR(%95CI)** |  | **No.*** | **HR(%95CI)** |  | **No.*** | **HR(%95CI)** |  |
| **Sex** |  |  |  |  |  |  |  |  |  |  |  |  |  |  |  |
| Women | 296 | 1.00 |  | 380 | 0.81 (0.69, 0.95) |  | 547 | 0.76 (0.65, 0.88) |  | 143 | 0.71 (0.58, 0.88) |  | 117 | 0.71 (0.56, 0.89) | 0.339 |
| Men | 244 | 1.00 |  | 462 | 0.94 (0.80, 1.10) |  | 826 | 0.84 (0.72, 0.98) |  | 222 | 0.79 (0.65, 0.96) |  | 198 | 0.76 (0.62, 0.92) |  |
| **Age (yr)** |  |  |  |  |  |  |  |  |  |  |  |  |  |  |  |
| <50 | 50 | 1.00 |  | 93 | 0.98 (0.69, 1.40) |  | 157 | 0.83 (0.59, 1.16) |  | 51 | 0.98 (0.65, 1.49) |  | 44 | 0.97 (0.63, 1.50) | 0.470 |
| 50-60 | 120 | 1.00 |  | 190 | 0.90 (0.71, 1.14) |  | 317 | 0.84 (0.67, 1.05) |  | 93 | 0.74 (0.56, 0.99) |  | 87 | 0.90 (0.67, 1.21) |  |
| ≥60 | 370 | 1.00 |  | 559 | 0.84 (0.73, 0.96) |  | 899 | 0.78 (0.68, 0.88) |  | 221 | 0.72 (0.60, 0.86) |  | 184 | 0.63 (0.52, 0.76) |  |
| **Area** |  |  |  |  |  |  |  |  |  |  |  |  |  |  |  |
| Urban | 52 | 1.00 |  | 86 | 0.85 (0.60, 1.20) |  | 248 | 0.84 (0.61, 1.16) |  | 32 | 0.82 (0.51, 1.31) |  | 89 | 1.05 (0.70, 1.58) | 0.042 |
| Rural | 488 | 1.00 |  | 756 | 0.87 (0.78, 0.98) |  | 1,125 | 0.79 (0.71, 0.89) |  | 333 | 0.75 (0.64, 0.87) |  | 226 | 0.68 (0.58, 0.80) |  |
| **Highest education level** |  |  |  |  |  |  |  |  |  |  |  |  |  |  |  |
| Primary school and below | 474 | 1.00 |  | 724 | 0.86 (0.77, 0.97) |  | 1,069 | 0.78 (0.69, 0.87) |  | 286 | 0.72 (0.62, 0.84) |  | 193 | 0.63 (0.53, 0.76) | 0.059 |
| Middle or high school | 66 | 1.00 |  | 114 | 0.87 (0.64, 1.18) |  | 285 | 0.84 (0.63, 1.11) |  | 73 | 0.79 (0.56, 1.13) |  | 111 | 0.97 (0.70, 1.35) |  |
| College and above | 0 | 1.00 |  | 4 | - |  | 19 | - |  | 6 | - |  | 11 | - |  |
| **Household income (yuan/yr)** |  |  |  |  |  |  |  |  |  |  |  |  |  |  |  |
| <10,000 | 396 | 1.00 |  | 546 | 0.85 (0.75, 0.98) |  | 672 | 0.79 (0.70, 0.91) |  | 134 | 0.61 (0.50, 0.75) |  | 141 | 0.60 (0.49, 0.73) | 0.012 |
| 10,000-19,999 | 106 | 1.00 |  | 169 | 0.86 (0.67, 1.10) |  | 405 | 0.85 (0.67, 1.07) |  | 130 | 0.94 (0.71, 1.24) |  | 93 | 0.84 (0.62, 1.13) |  |
| ≥20,000 | 38 | 1.00 |  | 127 | 0.94 (0.65, 1.37) |  | 296 | 0.79 (0.56, 1.12) |  | 101 | 0.87 (0.59, 1.29) |  | 81 | 1.07 (0.71, 1.62) |  |
| **Alcohol consumption** |  |  |  |  |  |  |  |  |  |  |  |  |  |  |  |
| Not current | 463 | 1.00 |  | 715 | 0.85 (0.75, 0.96) |  | 1,132 | 0.79 (0.71, 0.89) |  | 316 | 0.74 (0.64, 0.87) |  | 233 | 0.68 (0.58, 0.81) | 0.449 |
| Current | 77 | 1.00 |  | 127 | 0.96 (0.71, 1.28) |  | 241 | 0.78 (0.59, 1.02) |  | 49 | 0.74 (0.50, 1.08) |  | 82 | 0.89 (0.64, 1.24) |  |
| **Tobacco smoking** |  |  |  |  |  |  |  |  |  |  |  |  |  |  |  |
| Not current | 337 | 1.00 |  | 480 | 0.80 (0.70, 0.93) |  | 739 | 0.73 (0.63, 0.83) |  | 199 | 0.71 (0.59, 0.85) |  | 164 | 0.72 (0.59, 0.88) | 0.073 |
| Current | 203 | 1.00 |  | 362 | 0.98 (0.82, 1.17) |  | 634 | 0.91 (0.76, 1.07) |  | 166 | 0.81 (0.65, 1.01) |  | 151 | 0.75 (0.60, 0.94) |  |
| **Physical activity (MET-hr/day)** |  |  |  |  |  |  |  |  |  |  |  |  |  |  |  |
| Low | 277 | 1.00 |  | 386 | 0.82 (0.70, 0.96) |  | 770 | 0.80 (0.69, 0.93) |  | 182 | 0.74 (0.61, 0.90) |  | 168 | 0.68 (0.55, 0.83) | 0.689 |
| Middle | 161 | 1.00 |  | 271 | 0.91 (0.74, 1.11) |  | 338 | 0.74 (0.60, 0.90) |  | 94 | 0.64 (0.49, 0.85) |  | 86 | 0.73 (0.55, 0.96) |  |
| High | 102 | 1.00 |  | 185 | 0.93 (0.73, 1.20) |  | 265 | 0.84 (0.66, 1.07) |  | 89 | 0.90 (0.67, 1.21) |  | 61 | 0.84 (0.60, 1.18) |  |
| **Dietary pattern** |  |  |  |  |  |  |  |  |  |  |  |  |  |  |  |
| New affluence | 3 | 1.00 |  | 20 | 4.18 (1.23, 14.25) |  | 79 | 2.39 (0.74, 7.68) |  | 53 | 3.08 (0.95, 10.04) |  | 112 | 2.86 (0.90, 9.11) | 0.012 |
| Traditional southern | 286 | 1.00 |  | 402 | 0.81 (0.69, 0.95) |  | 863 | 0.77 (0.66, 0.89) |  | 177 | 0.68 (0.55, 0.84) |  | 89 | 0.78 (0.61, 0.99) |  |
| Traditional northern | 251 | 1.00 |  | 420 | 0.88 (0.75, 1.03) |  | 431 | 0.81 (0.69, 0.94) |  | 135 | 0.77 (0.62, 0.95) |  | 114 | 0.64 (0.51, 0.81) |  |
| **BMI (kg/m^2^)** |  |  |  |  |  |  |  |  |  |  |  |  |  |  |  |
| <24.0 | 341 | 1.00 |  | 601 | 0.92 (0.80, 1.06) |  | 932 | 0.81 (0.71, 0.92) |  | 262 | 0.77 (0.65, 0.91) |  | 210 | 0.77 (0.64, 0.92) | 0.480 |
| 24.0-28.0 | 137 | 1.00 |  | 179 | 0.81 (0.64, 1.02) |  | 334 | 0.79 (0.64, 0.98) |  | 79 | 0.71 (0.53, 0.96) |  | 84 | 0.72 (0.54, 0.97) |  |
| ≥28.0 | 62 | 1.00 |  | 62 | 0.70 (0.49, 1.01) |  | 107 | 0.71 (0.50, 1.00) |  | 24 | 0.69 (0.42, 1.15) |  | 21 | 0.47 (0.27, 0.81) |  |
| **Hypertension** |  |  |  |  |  |  |  |  |  |  |  |  |  |  |  |
| Yes | 126 | 1.00 |  | 195 | 0.80 (0.64, 1.00) |  | 364 | 0.78 (0.63, 0.97) |  | 103 | 0.74 (0.56, 0.97) |  | 110 | 0.82 (0.62, 1.08) | 0.347 |
| No | 414 | 1.00 |  | 647 | 0.89 (0.79, 1.01) |  | 1,009 | 0.80 (0.71, 0.90) |  | 262 | 0.76 (0.64, 0.89) |  | 205 | 0.69 (0.57, 0.82) |  |

HR indicates hazard ratios; CI confidence interval.

Stratified Cox proportional models were used with stratification on survey site and birth cohort (in 5-year intervals), except for subgroup and interaction analyses according to age at recruitment (only with stratification on survey site). Multivariate models were adjusted for age at recruitment (continuous) and sex (men or women); model 2: additionally included education level (no formal school, primary school, middle school, high school, college, or university or higher), household income (<2,500, 2,500-4,999, 5,000-9,999, 10,000-19,999, 20,000-34,999, or ≥35,000 yuan/year), marital status (married, widowed, divorced or separated, or never married), alcohol consumption (not weekly; ex-regular; not daily; daily consuming 1-15, 15-29, 30-59, or ≥60 g), tobacco smoking (never or occasional; former; current smoking with 1-14, 15-24, or ≥25 cigarettes/day), physical activity in MET-hours/day (continuous), BMI (continuous), waist to hip ratio (continuous), prevalent hypertension (presence or absence), use of aspirin (presence, absence, or unknown), family history of CVD (presence or absence); model 3: additionally included intake of multivitamin supplementation (presence or absence) and dietary pattern (new affluence, traditional northern, or traditional southern), as appropriate.

* No. of hemorrhagic stroke deaths.

† The tests for interaction were performed using likelihood ratio tests, which involved comparing models with and without interaction terms between the strata variable and egg consumption (as a multinomial variable).

**Supplemental Table S14. Subgroup analysis of associations between egg consumption and mortality from ischemic stroke according to potential baseline risk factors.**

| **Subgroups** | **Egg consumption** | | | | | | | | | | | | | | |
| --- | --- | --- | --- | --- | --- | --- | --- | --- | --- | --- | --- | --- | --- | --- | --- |
|  | **Never/rarely** | |  | **1-3 days/month** | |  | **1-3 d/week** | |  | **4-6 d/week** | |  | **7 d/week** | | ***P* for interaction†** |
|  | **No.*** | **HR** |  | **No.*** | **HR(%95CI)** |  | **No.*** | **HR(%95CI)** |  | **No.*** | **HR(%95CI)** |  | **No.*** | **HR(%95CI)** |  |
| **Sex** |  |  |  |  |  |  |  |  |  |  |  |  |  |  |  |
| Women | 53 | 1.00 |  | 103 | 1.18 (0.84, 1.65) |  | 185 | 1.31 (0.95, 1.81) |  | 25 | 0.89 (0.55, 1.46) |  | 48 | 1.20 (0.78, 1.84) | 0.161 |
| Men | 60 | 1.00 |  | 134 | 0.95 (0.70, 1.29) |  | 252 | 0.84 (0.63, 1.13) |  | 65 | 1.00 (0.69, 1.45) |  | 78 | 0.78 (0.55, 1.12) |  |
| **Age (yr)** |  |  |  |  |  |  |  |  |  |  |  |  |  |  |  |
| <50 | 8 | 1.00 |  | 12 | 0.68 (0.27, 1.69) |  | 31 | 0.82 (0.36, 1.87) |  | 11 | 1.37 (0.52, 3.60) |  | 5 | 0.52 (0.16, 1.72) | 0.438 |
| 50-60 | 17 | 1.00 |  | 44 | 1.41 (0.80, 2.48) |  | 78 | 1.20 (0.69, 2.07) |  | 13 | 0.84 (0.40, 1.79) |  | 17 | 0.88 (0.43, 1.80) |  |
| ≥60 | 88 | 1.00 |  | 181 | 1.02 (0.79, 1.32) |  | 328 | 1.02 (0.79, 1.30) |  | 66 | 0.99 (0.71, 1.39) |  | 104 | 0.97 (0.71, 1.33) |  |
| **Area** |  |  |  |  |  |  |  |  |  |  |  |  |  |  |  |
| Urban | 27 | 1.00 |  | 56 | 1.20 (0.76, 1.91) |  | 146 | 1.26 (0.81, 1.96) |  | 23 | 1.40 (0.77, 2.54) |  | 70 | 1.70 (1.00, 2.88) | 0.021 |
| Rural | 86 | 1.00 |  | 181 | 1.00 (0.77, 1.31) |  | 291 | 1.00 (0.78, 1.28) |  | 67 | 0.93 (0.67, 1.31) |  | 56 | 0.69 (0.49, 0.98) |  |
| **Highest education level** |  |  |  |  |  |  |  |  |  |  |  |  |  |  |  |
| Primary school and below | 98 | 1.00 |  | 196 | 1.00 (0.78, 1.27) |  | 339 | 1.02 (0.81, 1.30) |  | 64 | 0.91 (0.65, 1.27) |  | 77 | 0.83 (0.60, 1.14) | 0.517 |
| Middle or high school | 15 | 1.00 |  | 37 | 1.21 (0.66, 2.22) |  | 87 | 0.97 (0.55, 1.71) |  | 22 | 1.16 (0.58, 2.29) |  | 43 | 1.22 (0.65, 2.29) |  |
| College and above | 0 | 1.00 |  | 4 | - |  | 11 | - |  | 4 | - |  | 6 | - |  |
| **Household income (yuan/yr)** |  |  |  |  |  |  |  |  |  |  |  |  |  |  |  |
| <10,000 | 77 | 1.00 |  | 131 | 0.96 (0.72, 1.28) |  | 203 | 1.02 (0.77, 1.34) |  | 38 | 0.99 (0.66, 1.47) |  | 42 | 0.70 (0.47, 1.03) | 0.146 |
| 10,000-19,999 | 22 | 1.00 |  | 59 | 1.39 (0.85, 2.29) |  | 130 | 1.32 (0.82, 2.12) |  | 23 | 1.00 (0.54, 1.86) |  | 48 | 1.46 (0.85, 2.54) |  |
| ≥20,000 | 14 | 1.00 |  | 47 | 0.95 (0.52, 1.75) |  | 104 | 0.85 (0.48, 1.52) |  | 29 | 1.11 (0.57, 2.19) |  | 36 | 1.01 (0.51, 1.99) |  |
| **Alcohol consumption** |  |  |  |  |  |  |  |  |  |  |  |  |  |  |  |
| Not current | 102 | 1.00 |  | 198 | 1.00 (0.79, 1.28) |  | 374 | 1.06 (0.84, 1.33) |  | 75 | 0.95 (0.70, 1.30) |  | 106 | 0.94 (0.70, 1.26) | 0.211 |
| Current | 11 | 1.00 |  | 39 | 1.36 (0.68, 2.70) |  | 63 | 0.87 (0.44, 1.71) |  | 15 | 1.24 (0.54, 2.81) |  | 20 | 0.90 (0.40, 2.00) |  |
| **Tobacco smoking** |  |  |  |  |  |  |  |  |  |  |  |  |  |  |  |
| Not current | 64 | 1.00 |  | 140 | 1.18 (0.88, 1.60) |  | 240 | 1.14 (0.86, 1.53) |  | 46 | 1.08 (0.73, 1.61) |  | 68 | 1.07 (0.73, 1.55) | 0.833 |
| Current | 49 | 1.00 |  | 97 | 0.91 (0.64, 1.29) |  | 197 | 0.91 (0.66, 1.27) |  | 44 | 0.91 (0.60, 1.40) |  | 58 | 0.80 (0.53, 1.20) |  |
| **Physical activity (MET-hr/day)** |  |  |  |  |  |  |  |  |  |  |  |  |  |  |  |
| Low | 70 | 1.00 |  | 149 | 1.14 (0.85, 1.51) |  | 281 | 1.06 (0.81, 1.40) |  | 51 | 0.99 (0.68, 1.44) |  | 95 | 1.03 (0.74, 1.45) | 0.876 |
| Middle | 27 | 1.00 |  | 59 | 0.97 (0.61, 1.54) |  | 104 | 1.10 (0.70, 1.72) |  | 25 | 1.08 (0.60, 1.93) |  | 23 | 0.93 (0.51, 1.69) |  |
| High | 16 | 1.00 |  | 29 | 0.78 (0.41, 1.46) |  | 52 | 0.77 (0.42, 1.39) |  | 14 | 0.82 (0.38, 1.75) |  | 8 | 0.43 (0.17, 1.08) |  |
| **Dietary pattern** |  |  |  |  |  |  |  |  |  |  |  |  |  |  |  |
| New affluence | 3 | 1.00 |  | 8 | 1.37 (0.36, 5.20) |  | 52 | 1.18 (0.37, 3.81) |  | 22 | 1.39 (0.41, 4.70) |  | 67 | 1.49 (0.46, 4.80) | 0.211 |
| Traditional southern | 51 | 1.00 |  | 110 | 1.04 (0.74, 1.47) |  | 235 | 1.21 (0.87, 1.68) |  | 38 | 1.30 (0.82, 2.04) |  | 14 | 1.15 (0.63, 2.10) |  |
| Traditional northern | 59 | 1.00 |  | 119 | 1.05 (0.77, 1.44) |  | 150 | 0.95 (0.70, 1.29) |  | 30 | 0.83 (0.53, 1.29) |  | 45 | 0.69 (0.46, 1.03) |  |
| **BMI (kg/m^2^)** |  |  |  |  |  |  |  |  |  |  |  |  |  |  |  |
| <24.0 | 69 | 1.00 |  | 154 | 1.10 (0.82, 1.47) |  | 267 | 1.10 (0.83, 1.45) |  | 65 | 1.27 (0.89, 1.81) |  | 67 | 1.00 (0.70, 1.44) | 0.561 |
| 24.0-28.0 | 30 | 1.00 |  | 65 | 1.10 (0.71, 1.71) |  | 129 | 1.04 (0.68, 1.58) |  | 15 | 0.53 (0.28, 1.01) |  | 44 | 0.88 (0.52, 1.46) |  |
| ≥28.0 | 14 | 1.00 |  | 18 | 0.65 (0.32, 1.32) |  | 41 | 0.65 (0.35, 1.23) |  | 10 | 0.82 (0.34, 1.93) |  | 15 | 0.71 (0.32, 1.60) |  |
| **Hypertension** |  |  |  |  |  |  |  |  |  |  |  |  |  |  |  |
| Yes | 23 | 1.00 |  | 61 | 1.37 (0.84, 2.22) |  | 137 | 1.61 (1.02, 2.56) |  | 32 | 1.64 (0.93, 2.88) |  | 37 | 1.18 (0.67, 2.06) | 0.253 |
| No | 90 | 1.00 |  | 176 | 0.96 (0.74, 1.25) |  | 300 | 0.88 (0.68, 1.12) |  | 58 | 0.83 (0.58, 1.17) |  | 89 | 0.89 (0.64, 1.22) |  |

HR indicates hazard ratios; CI confidence interval.

Stratified Cox proportional models were used with stratification on survey site and birth cohort (in 5-year intervals), except for subgroup and interaction analyses according to age at recruitment (only with stratification on survey site). Multivariate models were adjusted for age at recruitment (continuous) and sex (men or women); model 2: additionally included education level (no formal school, primary school, middle school, high school, college, or university or higher), household income (<2,500, 2,500-4,999, 5,000-9,999, 10,000-19,999, 20,000-34,999, or ≥35,000 yuan/year), marital status (married, widowed, divorced or separated, or never married), alcohol consumption (not weekly; ex-regular; not daily; daily consuming 1-15, 15-29, 30-59, or ≥60 g), tobacco smoking (never or occasional; former; current smoking with 1-14, 15-24, or ≥25 cigarettes/day), physical activity in MET-hours/day (continuous), BMI (continuous), waist to hip ratio (continuous), prevalent hypertension (presence or absence), use of aspirin (presence, absence, or unknown), family history of CVD (presence or absence); model 3: additionally included intake of multivitamin supplementation (presence or absence) and dietary pattern (new affluence, traditional northern, or traditional southern), as appropriate.

* No. of ischemic stroke deaths.

† The tests for interaction were performed using likelihood ratio tests, which involved comparing models with and without interaction terms between the strata variable and egg consumption (as a multinomial variable) (as multinomial variables).

**Supplemental Table S15. Associations of egg consumption with risk of cardiovascular disease among 26,162 diabetic patients.**

| **Endpoints** | **Egg consumption** | | | | | **P for linear trend*** | **HR for 1 egg/week †** |
| --- | --- | --- | --- | --- | --- | --- | --- |
|  | **Never/rarely** | **1-3 days/month** | **1-3 d/week** | **4-6 d/week** | **7 d/week** |  |  |
| **CVD** |  |  |  |  |  |  |  |
| Cases | 781 | 1,520 | 3,857 | 783 | 1,944 |  |  |
| PYs | 15,942 | 35,775 | 86,691 | 17,975 | 37,523 |  |  |
| Cases/PYs (1/1000) | 49.0 | 42.5 | 44.5 | 43.6 | 51.8 |  |  |
| Model 1 | 1.00 | 0.91 (0.83, 0.99) | 0.90 (0.83, 0.97) | 0.83 (0.75, 0.92) | 0.82 (0.75, 0.90) | <0.001 | 0.95 (0.93, 0.98) |
| Model 2 | 1.00 | 0.92 (0.84, 1.01) | 0.93 (0.86, 1.01) | 0.88 (0.80, 0.98) | 0.86 (0.79, 0.94) | 0.002 | 0.96 (0.94, 0.99) |
| Model 3 | 1.00 | 0.98 (0.86, 1.11) | 0.95 (0.85, 1.07) | 0.87 (0.75, 1.02) | 0.88 (0.76, 1.01) | 0.025 | 0.96 (0.92, 1.00) |
| **IHD** |  |  |  |  |  |  |  |
| Cases | 297 | 556 | 1,584 | 345 | 996 |  |  |
| PYs | 17,396 | 38,999 | 95,047 | 19,532 | 41,217 |  |  |
| Cases/PYs (1/1000) | 17.1 | 14.3 | 16.7 | 17.7 | 24.2 |  |  |
| Model 1 | 1.00 | 0.95 (0.83, 1.10) | 0.93 (0.82, 1.06) | 0.89 (0.76, 1.05) | 0.90 (0.79, 1.04) | 0.188 | 0.98 (0.95, 1.01) |
| Model 2 | 1.00 | 0.97 (0.84, 1.12) | 0.95 (0.83, 1.08) | 0.93 (0.79, 1.09) | 0.92 (0.80, 1.06) | 0.252 | 0.98 (0.95, 1.01) |
| Model 3 | 1.00 | 0.98 (0.80, 1.21) | 0.93 (0.76, 1.12) | 0.88 (0.69, 1.14) | 0.94 (0.75, 1.17) | 0.591 | 0.99 (0.93, 1.04) |
| **MCE** |  |  |  |  |  |  |  |
| Cases | 86 | 151 | 397 | 73 | 238 |  |  |
| PYs | 18,202 | 40,613 | 99,883 | 20,689 | 44,570 |  |  |
| Cases/PYs (1/1000) | 4.7 | 3.7 | 4.0 | 3.5 | 5.3 |  |  |
| Model 1 | 1.00 | 0.95 (0.73, 1.25) | 0.93 (0.73, 1.19) | 0.72 (0.52, 0.99) | 0.87 (0.67, 1.13) | 0.119 | 0.96 (0.90, 1.02) |
| Model 2 | 1.00 | 0.98 (0.75, 1.29) | 0.99 (0.77, 1.26) | 0.80 (0.58, 1.11) | 0.93 (0.71, 1.22) | 0.280 | 0.97 (0.91, 1.04) |
| Model 3 | 1.00 | 0.93 (0.62, 1.40) | 0.97 (0.67, 1.42) | 0.54 (0.31, 0.95) | 0.76 (0.49, 1.20) | 0.048 | 0.91 (0.80, 1.02) |
| **Hemorrhagic stroke** |  |  |  |  |  |  |  |
| Cases | 94 | 167 | 291 | 83 | 81 |  |  |
| PYs | 18,171 | 40,493 | 99,855 | 20,651 | 44,652 |  |  |
| Cases/PYs (1/1000) | 5.2 | 4.1 | 2.9 | 4.0 | 1.8 |  |  |
| Model 1 | 1.00 | 0.92 (0.71, 1.20) | 0.73 (0.57, 0.94) | 0.81 (0.59, 1.10) | 0.44 (0.32, 0.60) | <0.001 | 0.79 (0.73, 0.86) |
| Model 2 | 1.00 | 0.96 (0.74, 1.24) | 0.79 (0.62, 1.01) | 0.93 (0.68, 1.27) | 0.50 (0.36, 0.69) | <0.001 | 0.83 (0.76, 0.90) |
| Model 3 | 1.00 | 0.93 (0.64, 1.35) | 0.82 (0.57, 1.18) | 1.12 (0.71, 1.77) | 0.73 (0.45, 1.18) | 0.565 | 0.94 (0.82, 1.07) |
| **Ischemic stroke** |  |  |  |  |  |  |  |
| Cases | 345 | 696 | 1,737 | 345 | 854 |  |  |
| PYs | 17,163 | 38,491 | 94,128 | 19,523 | 41,716 |  |  |
| Cases/PYs (1/1000) | 20.1 | 18.1 | 18.5 | 17.7 | 20.5 |  |  |
| Model 1 | 1.00 | 0.97 (0.85, 1.11) | 0.93 (0.82, 1.05) | 0.90 (0.77, 1.05) | 0.84 (0.73, 0.95) | 0.002 | 0.95 (0.92, 0.98) |
| Model 2 | 1.00 | 0.99 (0.87, 1.13) | 0.97 (0.86, 1.10) | 0.98 (0.84, 1.14) | 0.89 (0.78, 1.02) | 0.044 | 0.96 (0.93, 1.00) |
| Model 3 | 1.00 | 1.06 (0.87, 1.29) | 1.09 (0.91, 1.31) | 1.18 (0.93, 1.50) | 0.96 (0.77, 1.20) | 0.536 | 0.98 (0.92, 1.03) |

CVD: cardiovascular disease; IHD: ischemic heart disease; MCE: major coronary events.

Stratified Cox proportional models were used with stratification on survey site and birth cohort (in 5-year intervals). Multivariate models were adjusted for: model 1: age at recruitment (continuous) and sex (men or women); model 2: additionally included education level (no formal school, primary school, middle school, high school, college, or university or higher), household income (<2,500, 2,500-4,999, 5,000-9,999, 10,000-19,999, 20,000-34,999, or ≥35,000 yuan/year), marital status (married, widowed, divorced or separated, or never married), alcohol consumption (not weekly; ex-regular; not daily; daily consuming 1-15, 15-29, 30-59, or ≥60 g), tobacco smoking (never or occasional; former; current smoking with 1-14, 15-24, or ≥25 cigarettes/day), physical activity in MET-hours/day (continuous), BMI (continuous), waist to hip ratio (continuous), prevalent hypertension (presence or absence), use of aspirin (presence, absence, or unknown), family history of CVD (presence or absence); model 3: additionally included intake of multivitamin supplementation (presence or absence) and dietary pattern (new affluence, traditional northern, or traditional southern).

* Tests for linear trend were conducted by assigning 0, 0.5, 2.0, 5.0, 7.0 to the frequency levels from the lowest to the highest and treating the variable as a continuous variable in the Cox models.

† HRs for each 1 egg increment per week were calculated by using the usual amount in the multivariate Cox models

**Supplemental Table S16. Associations of egg consumption with mortality from cardiovascular disease among 26,162 diabetic patients.**

| **Endpoints** | **Egg consumption** | | | | | **P for linear trend*** | **HR for 1 egg/week †** |
| --- | --- | --- | --- | --- | --- | --- | --- |
|  | **Never/rarely** | **1-3 days/month** | **1-3 d/week** | **4-6 d/week** | **7 d/week** |  |  |
| **PYs** | 18,294 | 40,722 | 100,296 | 20,755 | 44,797 |  |  |
| **CVD** |  |  |  |  |  |  |  |
| Deaths | 163 | 320 | 649 | 128 | 279 |  |  |
| Deaths/PYs (1/1000) | 8.9 | 7.9 | 6.5 | 6.2 | 6.2 |  |  |
| Model 1 | 1.00 | 1.06 (0.88, 1.29) | 0.90 (0.75, 1.08) | 0.76 (0.60, 0.97) | 0.67 (0.55, 0.83) | <0.001 | 0.87 (0.82, 0.91) |
| Model 2 | 1.00 | 1.10 (0.90, 1.33) | 0.99 (0.82, 1.18) | 0.90 (0.70, 1.14) | 0.77 (0.63, 0.95) | <0.001 | 0.90 (0.85, 0.95) |
| Model 3 | 1.00 | 1.11 (0.83, 1.47) | 1.12 (0.85, 1.46) | 0.85 (0.58, 1.24) | 0.78 (0.56, 1.10) | 0.012 | 0.90 (0.82, 0.99) |
| **IHD** |  |  |  |  |  |  |  |
| Deaths | 57 | 109 | 258 | 46 | 160 |  |  |
| Deaths/PYs (1/1000) | 3.1 | 2.7 | 2.6 | 2.2 | 3.6 |  |  |
| Model 1 | 1.00 | 1.09 (0.79, 1.51) | 0.92 (0.68, 1.25) | 0.68 (0.46, 1.02) | 0.82 (0.60, 1.14) | 0.026 | 0.92 (0.85, 1.00) |
| Model 2 | 1.00 | 1.13 (0.81, 1.56) | 1.00 (0.74, 1.35) | 0.80 (0.53, 1.20) | 0.92 (0.66, 1.29) | 0.152 | 0.95 (0.88, 1.03) |
| Model 3 | 1.00 | 1.08 (0.67, 1.74) | 1.06 (0.67, 1.66) | 0.55 (0.28, 1.09) | 0.83 (0.48, 1.42) | 0.089 | 0.91 (0.79, 1.05) |
| **Hemorrhagic stroke** |  |  |  |  |  |  |  |
| Deaths | 55 | 106 | 156 | 43 | 44 |  |  |
| Deaths/PYs (1/1000) | 3.0 | 2.6 | 1.6 | 2.1 | 1.0 |  |  |
| Model 1 | 1.00 | 1.08 (0.77, 1.50) | 0.82 (0.59, 1.14) | 0.79 (0.52, 1.20) | 0.52 (0.34, 0.79) | <0.001 | 0.81 (0.72, 0.90) |
| Model 2 | 1.00 | 1.10 (0.79, 1.54) | 0.91 (0.65, 1.26) | 0.95 (0.62, 1.45) | 0.64 (0.42, 0.97) | 0.014 | 0.86 (0.77, 0.97) |
| Model 3 | 1.00 | 1.04 (0.66, 1.64) | 0.92 (0.59, 1.44) | 1.04 (0.58, 1.88) | 0.80 (0.44, 1.47) | 0.516 | 0.94 (0.79, 1.10) |
| **Ischemic stroke** |  |  |  |  |  |  |  |
| Deaths | 27 | 40 | 88 | 12 | 33 |  |  |
| Deaths/PYs (1/1000) | 1.5 | 1.0 | 0.9 | 0.6 | 0.7 |  |  |
| Model 1 | 1.00 | 0.70 (0.43, 1.15) | 0.65 (0.42, 1.03) | 0.45 (0.22, 0.90) | 0.42 (0.24, 0.72) | 0.003 | 0.79 (0.67, 0.92) |
| Model 2 | 1.00 | 0.77 (0.47, 1.27) | 0.77 (0.49, 1.22) | 0.60 (0.30, 1.22) | 0.50 (0.28, 0.88) | 0.017 | 0.82 (0.71, 0.96) |
| Model 3 | 1.00 | 1.01 (0.44, 2.33) | 1.11 (0.50, 2.45) | 0.47 (0.12, 1.87) | 0.66 (0.24, 1.83) | 0.191 | 0.86 (0.65, 1.13) |

CVD: cardiovascular disease; IHD: ischemic heart disease.

Stratified Cox proportional models were used with stratification on survey site and birth cohort (in 5-year intervals). Multivariate models were adjusted for: model 1: age at recruitment (continuous) and sex (men or women); model 2: additionally included education level (no formal school, primary school, middle school, high school, college, or university or higher), household income (<2,500, 2,500-4,999, 5,000-9,999, 10,000-19,999, 20,000-34,999, or ≥35,000 yuan/year), marital status (married, widowed, divorced or separated, or never married), alcohol consumption (not weekly; ex-regular; not daily; daily consuming 1-15, 15-29, 30-59, or ≥60 g), tobacco smoking (never or occasional; former; current smoking with 1-14, 15-24, or ≥25 cigarettes/day), physical activity in MET-hours/day (continuous), BMI (continuous), waist to hip ratio (continuous), prevalent hypertension (presence or absence), use of aspirin (presence, absence, or unknown), family history of CVD (presence or absence); model 3: additionally included intake of multivitamin supplementation (presence or absence) and dietary pattern (new affluence, traditional northern, or traditional southern).

* Tests for linear trend were conducted by assigning 0, 0.5, 2.0, 5.0, 7.0 to the frequency levels from the lowest to the highest and treating the variable as a continuous variable in the Cox models.

† HRs for each 1 egg increment per week were calculated by using the usual amount in the multivariate Cox models.
